# *Ligilactobacillus agilis* W70: a probiotic with capacity to enhance ammonia assimilation in the rumen

**DOI:** 10.3389/fmicb.2024.1498109

**Published:** 2025-01-17

**Authors:** Xiaowei Duan, Stafford Vigors, Rui Ma, Lu Ma, Jingang Gu, Dengpan Bu

**Affiliations:** ^1^State Key Laboratory of Animal Nutrition and Feeding, Institute of Animal Science, Chinese Academy of Agricultural Sciences, Beijing, China; ^2^School of Agriculture and Food Science, University College Dublin, Dublin, Ireland; ^3^Institute of Agricultural Resources and Regional Planning, Chinese Academy of Agricultural Sciences, Beijing, China; ^4^National Center of Technology Innovation for Dairy, Inner Mongolia, China

**Keywords:** *Ligilactobacillus agilis*, NH_3_N, rumen fermentation, screening, functional, physicochemical characterization

## Abstract

Ruminants excrete both undigested and excess consumed nitrogen (N) through feces and urine, which results in the waste of feed protein and environmental pollution. This study aims to screen bacteria to identify strains with potential to improve nitrogen utilization in the rumen. In a tube screening test, when 30 mmol/L ammonium sulfate was added as the only nitrogen source in liquid medium, among 115 bacterial strains belonging to *Bacillus* and lactic-acid bacteria, 10 strains with the highest growth (OD_600nm_ level) in comparison to the other evaluated strains were identified, and of these, *Ligilactobacillus agilis* W70 and *Limosilactobacillus fermentum* M50, had the highest ammonia nitrogen utilizing capabilities of ammonium sulfate (32.09 and 40.64%, respectively). A subsequent *in vitro* fermentation experiment was conducted with the diet consisting of 0.5 g TMR, 50 mL buffer solution, and 25 mL fresh rumen fluid per serum bottle which was incubated at 39°C for 24 h. The experiment consisted of 3 treatments, CTL, *L. agilis* W70 (1 × 10^9^ cfu/mL), and *L. fermentum* M50 (1 × 10^9^ cfu/mL), each treatment had 6 replicates, repeated across 3 batches. The addition of *L. agilis* W70 decreased NH_3_N (*p* < 0.01), the ratio of acetate to propionate (A:P) (*p* < 0.01), and increased the yields of microbial protein (MCP) (*p* < 0.01) and enzymatic activities of glutamate dehydrogenase (GDH) (*p* < 0.05) and glutamine synthetase (GS) (*p* < 0.05). Further genome analysis revealed that the *L. agilis* W70 harbors the glutamate dehydrogenase and glutamine synthetase-encoding genes *gdhA* and *glnA* that play key roles in ammonia utilization. This study identified *L. agilis* W70 as a strain which exhibits high NH_3_N utilization capability and enhances ammonia assimilation *in vitro*. The strain was further characterized to elucidate this ammonia assimilation potential. Further studies will be conducted to develop strain *L. agilis* W70 as a new feed additive to improve the nitrogen utilization efficiency in ruminant animals.

## 1 Introduction

Ruminants have low nitrogen utilization efficiency of around 25% due to their unique digestive tract structure (Bach et al., 2005; Lavery and Ferris, 2021), where rumen microorganisms play a role in nitrogen metabolism (Liu et al., 2023), with some fermented protein converted into ammonia (Eschenlauer et al., 2002). If unutilized this ammonia excreted in urine and feces leads to both a waste of protein resources and environmental pollution, with nitrogen in urine being especially harmful due to its high potential for volatilization (Qi et al., 2023).

Supplementation of direct fed microbials (DFMs) in feed is an environmentally friendly, cost-effective, and biologically safe practice to reduce ruminal ammonia emissions and to increase nitrogen utilization efficiency (Callaway et al., 2023). Moreover, DFMs have potential to stabilize ruminal microbiota, improve nitrogen flow to the lower digestive tract, and increase milk protein yield (Karimi, 2022). *Bacillus* species are non-toxic, spore-forming bacteria that can colonize the intestinal tract; they are aerobic and well-suited to maintain viability during industrial production and storage (Cappellozza et al., 2023). Similarly, lactic acid bacteria (LAB) are non-toxic, produce lactic acid to help regulate rumen pH, and are facultative anaerobes, making them highly compatible with production and storage requirements (Kesen and Aiyegoro, 2018). Among them, ammonia utilizing bacteria assimilate ammonia nitrogen as a nitrogen source to produce microbial protein (MCP) in the rumen, (Zurak et al., 2023), which can be transformed to amino acids in the small intestine and transferred to the mammary gland to produce milk protein (Griswold et al., 2003). In view of the importance of improving animal productivity, it is a priority to screen and identify ammonia utilizing bacteria to regulate rumen fermentation to reduce rumen ammonia nitrogen emissions and enhance MCP production.

Studies on the isolation and functional characterization of rumen-originated ammonia nitrogen utilizing bacteria so far have been scarce. The advantage of this study is that our previous research cultured many rumen-derived strains using the culturomics method. From these, we intend to screen ammonia utilizing strains in this study, ensuring they are well-adapted to the rumen environment (Li et al., 2022). Screening ammonia-utilizing bacteria with ammonium sulfate as the sole nitrogen source is effective because it ensures that only bacteria proficient in ammonia metabolism will grow (Liu et al., 2020). *In vitro* studies offer a controlled setting to assess the impact of DFMs on rumen fermentation, which is crucial for evaluating their efficacy before applying them *in vivo* (Mi et al., 2019). Additionally, genetic analysis can offer insights into the specific genes and metabolic pathways involved in ammonia assimilation (Kim et al., 2014). This study aimed to screen rumen bacteria for higher ammonia nitrogen utilizing capability, evaluate their potential to reduce ammonia nitrogen emission and improve MCP production in the rumen, and explore potential genomic mechanisms of the identified ammonia nitrogen utilizing bacteria.

## 2 Materials and methods

### 2.1 Screening and evaluation of rumen bacteria in medium

#### 2.1.1 Primary screening test

One hundred and fifteen strains derived from rumen contents, belonging to *Bacillus* and lactic-acid bacteria (*Ligilactobacillus, Limosilactobacillus, Lacticaseibacillus, Weissella, Pediococcus,* and *Enterococcus*) identified previously (Duan et al., 2023) and conserved in the Ruminant Nutrition Laboratory in the Institute of Animal Science, Chinese Academy of Agricultural Sciences were selected for the primary screening test. Of these, 26 strains grown under facultative anaerobic gas conditions and 89 strains grown in strictly anaerobic gas conditions were inoculated at the ratio of 10% (v/v) into a 50 mL flask containing 15 mL liquid medium (modified MRS for lactic-acid bacteria and modified LB for *Bacillus* respectively). For facultative anaerobic gas conditions, the liquid medium was dispensed into screw-cap tubes sealed with butyl rubber stoppers, autoclaved, inoculated with bacteria, and then cultivated in a microbiological incubator under static conditions. For strictly anaerobic gas condition, the liquid medium was dispensed into screw-cap tubes sealed with butyl rubber stoppers, deoxygenated by gassing with 100% N_2_, autoclaved, inoculated with bacteria, and then cultivated in an anaerobic workstation under static conditions. The gas composition of the anaerobic workstation (Thermo 1,029, Waltham, USA) consisted of 85% N_2_, 10% H_2_, and 5% CO_2_. An oxygen indicator pack (Mitsubishi Gas Chemical Company, Tokyo, Japan) was used to monitor oxygen levels in the anaerobic workstation, displaying blue at oxygen levels above 0.5%, an intermediate pink-blue at 0.1–0.5%, and pink below 0.1%, indicating a stable anaerobic environment when consistently pink. MRS medium contains the following components: 10 g/L peptone, 8 g/L meat extract, 4 g/L yeast extract, 20 g/L C_6_H_12_O_6_, 2 g/L K_2_HPO_4_, 5 g/L sodium acetate, 2 g/L triammonium citrate, 0.2 g/L MgSO_4_ 7H_2_O, 0.05 g/L MnSO_4_ 4H_2_O, and 1 mL/L Tween 80. The modified MRS medium was prepared by replacing the nitrogen sources—peptone, meat extract, and yeast extract—with ammonium sulfate as the sole nitrogen source. The modified LB medium was prepared following the method of Liu et al. (2020), also using ammonium sulfate as the sole nitrogen source. Ammonium sulfate concentrations were set at 5, 10, 15, 20, 25, and 30 mmol/L. The strains were incubated at 39°C for 72 h, and the optical density (OD) values at 600 nm were determined.

#### 2.1.2 Comparison of ammonia utilizing capability

Based on the screening results of the primary test, 10 strains with the highest OD_600nm_ levels on 30 mmol/L ammonium sulfate were selected for the secondary screening test. The bacteria were inoculated and cultured as described above. The OD_600nm_ was monitored at 0 h, 24 h, 48 h, and 72 h. In addition, the NH_3_N contents in the supernatant and MCP yields were determined using an automated phenol-hypochlorite method (Broderick and Kang, 1980) and Folin phenol method (Makkar et al., 1982) respectively.

#### 2.1.3 Strain growth curve and microbial protein synthesis under various ammonium sulfate concentrations

Based on the results of the secondary screening test, *Ligilactobacillus agilis* W70 and *Limosillactobacillus fermentum* M50 were cultured in modified MRS medium containing 5, 10, 15, 20, 25, 30 mmol/L ammonium sulfate as the sole nitrogen source for 72 h. The OD_600nm_ were determined at 0 h, 24 h, 48 h, and 72 h, and the NH_3_N contents and MCP yields were determined at 0 h and 72 h as described above.

#### 2.1.4 Primary screening of cellulase, amylase, protease, and lipase activities

The W70 and M50 strains were cultured separately on carboxymethyl cellulose (CMC) agar, starch agar, skim milk agar, and Tween 80 agar media to assess their cellulolytic, amylolytic, proteolytic, and lipolytic activities, respectively, for 72 h at 39°C (Masi et al., 2023). The concentration and enzymatic activity produced were indicated by the diameter of the hydrolysis zone in each specific medium.

### 2.2 Evaluation of bacteria using *in vitro* rumen fermentation

#### 2.2.1 Preparation of rumen fluid solution and diets

All animal procedures were approved by the Institutional Animal Care and Use Committee of the Institute of Animal Science, Chinese Academy of Agricultural Sciences and complied with the protocol IAS2022-91. Three lactating Holstein cows with the same parity, health condition, and fitted with a permanent rumen fistula were used as rumen fluid donors. The cows were fed total mixed ration (TMR) twice a day (7:00 and 19:00) and drank freely. The Total Mixed Ration (TMR) diet consisting of corn silage (57.61%), alfalfa meal (12.39%), steam-flaked corn grain (8.91%), grind corn grain (7.61%), soybean meal (6.74%), soybean hulls (2.61%), VitTM premix (1.41%), corn gluten meal (1.09%), molasses (0.65%), cottonseed (0.43%), sodium bicarbonate (0.33%), and Bergafat (0.22%). The nutrient composition of the diet is as follows: DM 43.1% as fed, CP 14.7% DM, Ash 5.5% DM, ADF 22.5% DM, NDF 37.2% DM, NE_L_ 1.74 Mcal/kg. The rumen fluid was collected 2 h after morning feeding, mixed and put into an autoclaved thermos bottle, and brought back to the laboratory. Half a gram of TMR was put into each serum bottle as a fermentation substrate. The ingredient and chemical composition of the diets *in vitro* rumen fermentation are shown in Table 1. Under anaerobic and sterile conditions, 25 mL rumen fluid was filtered through four layers of cheesecloth, and then diluted with 50 mL of modified Menke and Steingass buffer solution (Menke, 1988).

**Table 1:** Ingredients of the experimental diets based on DM (%).

Item	TMR
Ingredient, % of DM	
Corn silage	32.58
Corn grain, steam-flaked	28.71
Soybean meal	17.53
Alfalfa meal	13.14
Dairy cow premix	2.96
Expanded soybean	2.18
BergaFat	1.60
Corn gluten meal	1.31
Nutrient composition, % of DM	
DM, % as fed	81.73
CP	19.61
EE	3.26
Ash	5.66
NDF	23.02
ADF	17.54
NE_L_^1^, Mcal/kg	1.44

1 NE_L_, the net energy for lactation derived from CPM-Dairy 3.8.0.1 (collaboratively developed by Cornell University, the University of Pennsylvania, and the Miner Institute), whereas other parameters are obtained through empirical measurements.

#### 2.2.2 *In vitro* rumen fermentation

Inocula of the W70 and M50 strains were prepared in the form of freeze-dried powder and added to serum bottles at a final concentration of 1 × 10^9^ cfu/g and cultured at 39°C for 24 h. The experiment contained three treatment groups: CTL group (control), Group W70 (1 × 10^9^ cfu/g W70), and Group M50 (1 × 10^9^ cfu/g M50). The choice of 1 × 10^9^ cfu for *in vitro* rumen fermentation was based on prior literature regarding the effects of direct fed microbials (DFM) on in vitro fermentation (Opsi et al., 2012; Cappellozza et al., 2023; Fitriyah et al., 2024). The *in vitro* fermentation was conducted using 6 replicates per treatment and the experiment was repeated 3 times giving 18 replicates per treatment in total.

#### 2.2.3 Sample collection and analysis

After 24 h fermentation, the gas volume of each serum bottle was measured, and the pH values of fermentation mixtures were determined immediately after uncapping. The fermentation mixtures were then collected and filtered through nylon bags. For NH_3_N and volatile fatty acid (VFA) analyses, 25% meta-phosphoric acid was added to the fermentation supernatant at the ratio of 20% (v/v), centrifuged at 10,000 × *g* at 4°C for 10 min, and stored at −20°C. The VFA contents were determined by gas chromatography as described previously (Stewart and Duncan, 1985). The NH_3_N contents and MCP yields were determined as described above. Enzymatic activities of glutamate dehydrogenase (GDH) and glutamine synthetase (GS) were assessed colourimetrically using an assay kit (Beijing Boxbio Science & Technology Co. Ltd., Beijing, China) according to the manufacturer’s protocol. The residue DM (dry matter) was estimated after drying and compared with initial DM to calculate the dry matter digestibility (DMD) as described previously (Van Soest and Robertson, 1985).

### 2.3 Phenotype and genome analysis of *Ligilactobacillus agilis* W70

#### 2.3.1 Phenotypic characterization and identification

Gram staining of the W70 strain was performed using the modified Hucker’s method (Hucker and Conn, 1923). Plate streaking of the W70 strain was performed on an MRS agar plate. The growth of the W70 strain under facultative anaerobic conditions at 39°C was monitored for 48 h. To confirm the identity of the W70 strain, genomic DNA was extracted using a Bacterial DNA kit (Solarbio, Beijing, China) according to the manufacturer’s instructions. The 16S rRNA gene fragment of the W70 strain was amplified from genomic DNA using the universal primers 27F (5’-AGAGTTTGATCMTGGCTCAG-3′) and 1492R (5’-TACGGYTACCTTGTTACGACTT-3′) (Frank et al., 2008). The PCR reaction was performed with a MyCycler Thermal Cycler (Bio-Rad, Hercules, CA, USA) in a volume of 20 μL containing 10 μL of 2 × TaqPCR StarMix (Genstar, Beijing, China), 8.2 μL of ddH_2_O, 1 μL of bacterial DNA template, and 0.4 μL of each primer. The cycling conditions were as follows: 2 min at 94°C and 30 cycles of 94°C for 30 s, 60°C for 30 s, and 72°C for 1 min, with a final extension at 72°C for 10 min. The PCR products were sequenced using a Sanger sequencer (Applied Biosystems 3730XL, Foster City, CA, USA). The 16S rRNA sequence of the W70 strain was aligned with those of *Ligilactobacillus* type species using Clustal W, and a phylogenetic analysis was performed using the maximum likelihood method in MEGA 6^1^ (Li et al., 2020). The ribosomal protein of the W70 strain was extracted in 1 mL of lysis buffer (70% formic acid) and 1 mL of matrix solution (saturated *α*-cyano acid-4-hydroxycinnamic in 50% acetonitrile and 2.5% trifluoroacetic acid) and identified by MALDI-TOF MS using the EXS3000 system (Zybio Inc., Chongqing, China). The spectrum was compared with those of known bacteria in the database. Isolates were accurately identified to the species and genus levels with the score values of greater than 1.9 and 1.7–1.9.

#### 2.3.2 Genomic DNA extraction

A single colony of the W70 strain on the MRS agar plate was inoculated into 2 mL of MRS medium and grown at 39°C for approximately 15 h (maximum growth). The cell biomass was harvested by 10 min centrifugation at 12,000 × *g*. Genomic DNA was then extracted using the Bacterial/fungal DNA extraction kit (Majorbio, Shanghai, China) according to the manufacturer’s protocol. Genomic DNA was gel-purified and quantified for further study.

#### 2.3.3 Library construction and genome sequencing

The whole genome sequencing and assembly were carried out by the Majorbio Bio-pharm Technology Co., Ltd., Shanghai, China. Genomic DNA was sequenced using the combination of PacBio Sequel IIe and Illumina sequencing platforms. For Illumina sequencing, genomic DNA was sheared into 400–500 bp fragments using a Covaris M220 Focused Acoustic Shearer following the manufacturer’s protocol. Illumina sequencing libraries were prepared from the sheared fragments using the NEXTFLEX Rapid DNA-Seq Kit. Briefly, the 5′ prime ends were first end-repaired and phosphorylated; the 3′ ends were then A-tailed and ligated to sequencing adapters; and the adapters-ligated products were enriched using PCR. The libraries were paired-end sequenced (2 × 150 bp) on Illumina Novaseq 6,000. For PacBio sequencing, genomic DNA was fragmented at ~10 kb. The fragments were then purified, end-repaired, and ligated with SMRT bell sequencing adapters following manufacturer’s recommendations (Pacific Biosciences, Menlo Park, CA, USA). The PacBio library was p sequenced on one SMRT cell using standard methods.

#### 2.3.4 Genome assembly and annotation

The raw Illumina sequencing reads generated from the paired-end library were subjected to quality-filtering using fastp v0.23.0. High-fidelity (HiFi) sequencing reads were generated from the PacBio platform for further analysis. Clean short reads and HiFi reads were assembled to construct the complete genome using Unicycle v0.4.8 (Wick et al., 2017) and Pilon v1.22 to polish the assembly using short-read alignments, reducing the error rate. The coding sequences (CDs) of chromosome and plasmid were predicted using the Glimmer or Prodigal v2.6.3 (Hyatt et al., 2010) and GeneMarkS (Besemer and Borodovsky, 2005) respectively. tRNA-scan-SE (v2.0) (Chan and Lowe, 2019) and Barrnap v0.9^2^ were used for tRNA and rRNA prediction, respectively. The CDs were annotated from NR, Swiss-Prot, Pfam, GO, COG, and KEGG database using sequence alignment tools such as BLAST, Diamond and HMMER. Briefly, each set of query proteins were aligned with the databases, and annotations of best-matched subjects (*e*-value <10^−5^) were obtained for gene annotation. Biosynthetic gene clusters (BGCs) of secondary metabolites were identified by the antiSMASH v5.1.2.

#### 2.3.5 The homology models of deduced protein of *gdhA* and *glnA*

The deduced protein sequences of *gdhA* and *glnA* were blasted against the databases of Swiss-prot (Uniprot)^3^ and PDB^4^. Homology-modeled using the Swiss Model^5^ with the PDB templates 5GUD for *gdhA* and 7TDP for *glnA*, respectively. Alignment with protein structure-resolved homologs of >50% identities using ClustalW and identification of catalytic domains, catalytic residues, and substrate binding sites using ESPript^6^.

#### 2.3.6 Gene expression analysis of *gdhA* and *glnA* by quantitative real-time PCR

To evalute the gene expression of *gdhA* and *glnA* between the different nitrogen concentrations, quantitative real-time PCR (RT-qPCR) analyses were performed. The total RNA was extracted from W70 grown in 5 mmol/L and 30 mmol/L ammonium sulfate as the sole nitrogen source modified MRS medium by SteadyPure Universal RNA Extraction Kit (Accurate Biotechnology Co. Ltd., Changsha, Hunan, China) and reverse transcribed into cDNA by using cDNA Synthesis Kit (Beijing Solarbio Science & Technology Co. Ltd., Beijing, China). Quantitative PCR was performed using SYBR Green (Thermo Fisher Scientific, Waltham, MA, USA) by using a Real-Time PCR System (Applied Biosystems, Carlsbad, CA, USA) in a 10 μL mixture containing cDNA template 1 μL, forward and reverse primer 0.5 μL each, SYBR Green qPCR Mix 5 μL, and ddH_2_O 3.5 μL. The PCR amplification was performed under the following conditions: 95°C for 2 min, followed by 40 cycles at 94°C for 15 s, and 60°C for 60 s of annealing extension, finally at 95°C for 15 s, 60°C for 60 s and 95°C for 15 s, for dissociation curve analysis. Specific qPCR primers were designed by Primer-BLAST^7^ based on the *gdhA* and *glnA* sequences of W70 and synthesized by BGI, Shenzhen, China (Supplementary Table S1). The relative expression ratio of *gdhA* and *glnA* genes versus the 16S rDNA gene was calculated using the 2^-ΔΔCT^ method, and all data were given in terms of relative mRNA expression (Liu et al., 2020).

### 2.4 Data analysis and availability

The OD_600nm_, MCP, NH_3_N and RT-qPCR data of 10 strains were analyzed by one-way ANOVA in SAS 9.4 (SAS Institute, Cary, NC, USA). The results are presented as histograms made using Origin (Version 8.0.0). Least square means were reported, and statistical significance was set at *p* < 0.05.

The data were analyzed using the PROC GLM procedure of SAS 9.4 (SAS Institute, Cary, NC, USA) to compare the means of gas, pH, total VFA, acetate, propionate, isobutyrate, butyrate, isovalerate, valerate, the ratio of acetate and propionate (A:P), DMD, NH_3_N, MCP, GDH, and GS *in vitro* rumen fermentation using the W70 and M50 strains. Treatment was set as the fixed effect and batch was set as a random effect in the model. Differences were tested by the tukey method. Statistical significance was declared at *p* < 0.05.

## 3 Results

### 3.1 Screening for ammonia nitrogen utilizing strains

A total of 115 rumen bacteria were initially screened for their ability to utilize ammonia nitrogen with ammonium sulfate as the sole nitrogen source. In facultative anaerobic gas conditions, *Bacillus safenisis* B55, *Ligilactobacillus agilis* W70, *Bacillus safenisis* B52, *Bacillus safenisis* F79, and *Weissella confuse* W26 were the strains with the highest growth (OD_600nm_ level) in 30 mmol/L ammonium sulfate (Supplementary Figure S1). In strictly anaerobic gas conditions, *Enterococcus faecium* F42, *Limosilactobacillus fermentum* M50, *Enterococcus lactis* M21, *Enterococcus faecium* B2, and *Lactobacillus salivarius* X36 showed the highest growth (OD_600nm_ level) in 30 mmol/L ammonia nitrogen (Supplementary Figure S2).

**Figure 1:**
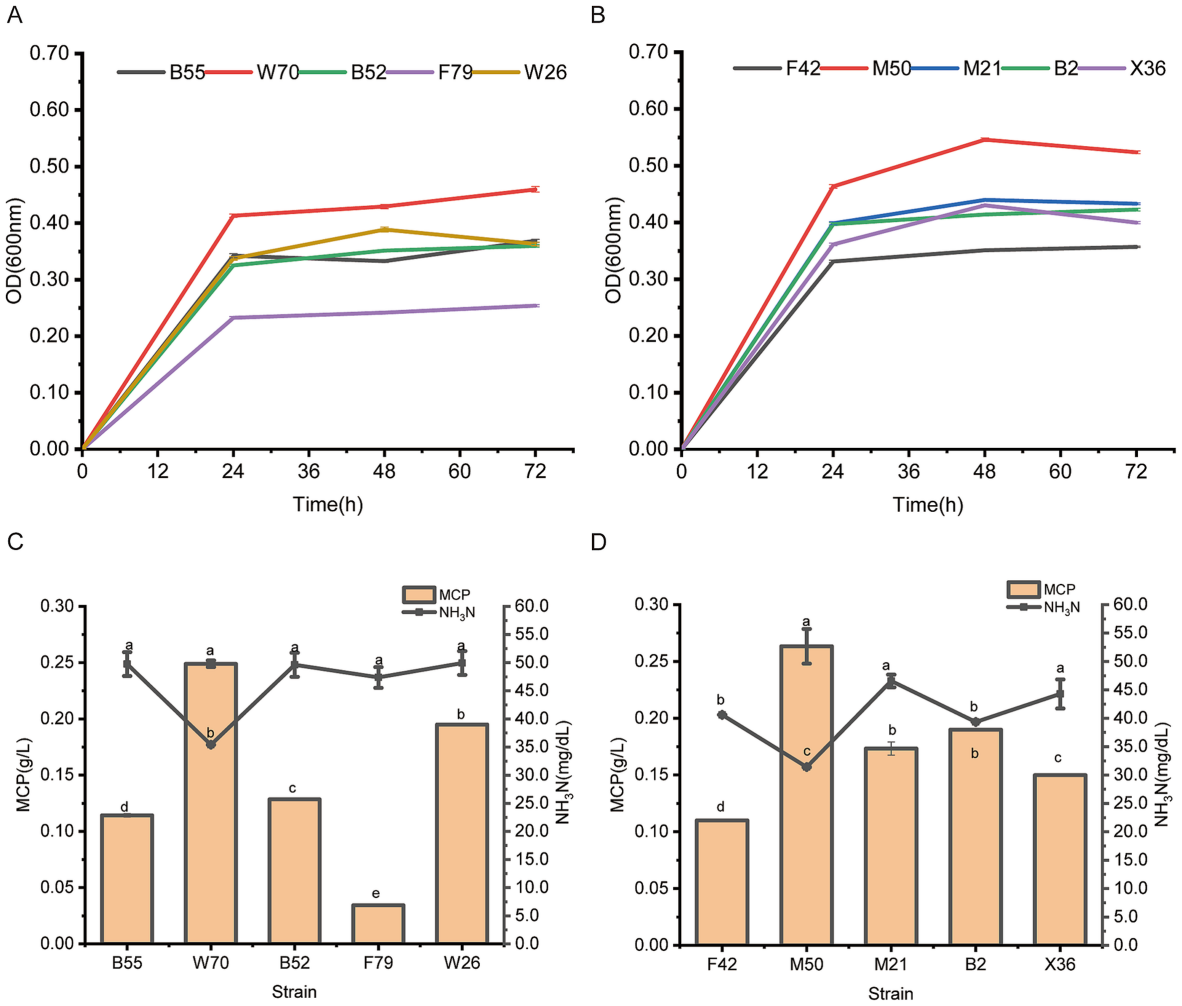
Growth curve and ammonia nitrogen utilizing capacities of *Bacillus safenisis* B55, *Lactobacillus agilis* W70, *Bacillus safenisis* B52, *Bacillus safenisis* F79, *Weissella confuse* W26, *Enterococcus faecium* F42, *Lactobacillus fermentum* M50, *Enterococcus lactis* M21, *Enterococcus faecium* B2, and *Lactobacillus salivarius* X36 with 30 mmol/L ammonium sulfate as the sole nitrogen source. **(A)** Growth curves of five strains under facultative anaerobic conditions. **(B)** Growth curves of five strains under strictly anaerobic conditions. **(C)** Ammonia nitrogen utilizing capacities of five strains under facultative anaerobic conditions. **(D)** Ammonia nitrogen utilizing capacities of five strains under strictly anaerobic conditions. Data are shown as mean ± standard deviation of three experiments. Different letters refer to statistically significant differences at *p* < 0.05.

To further analyze these strain’s ammonia nitrogen utilizing capacity, a secondary screening test, using 30 mmol/L ammonium sulfate as the sole nitrogen source was conducted. *L. agilis* W70 and *L. fermentum* M50 outperformed the other four tested strains following 72 h incubation under facultative anaerobic and strictly anaerobic gas conditions, respectively, (Figures 1A,B), and showed greater capabilities to reduce ammonia nitrogen and produce MCP (Figures 1C,D).

**Figure 2:**
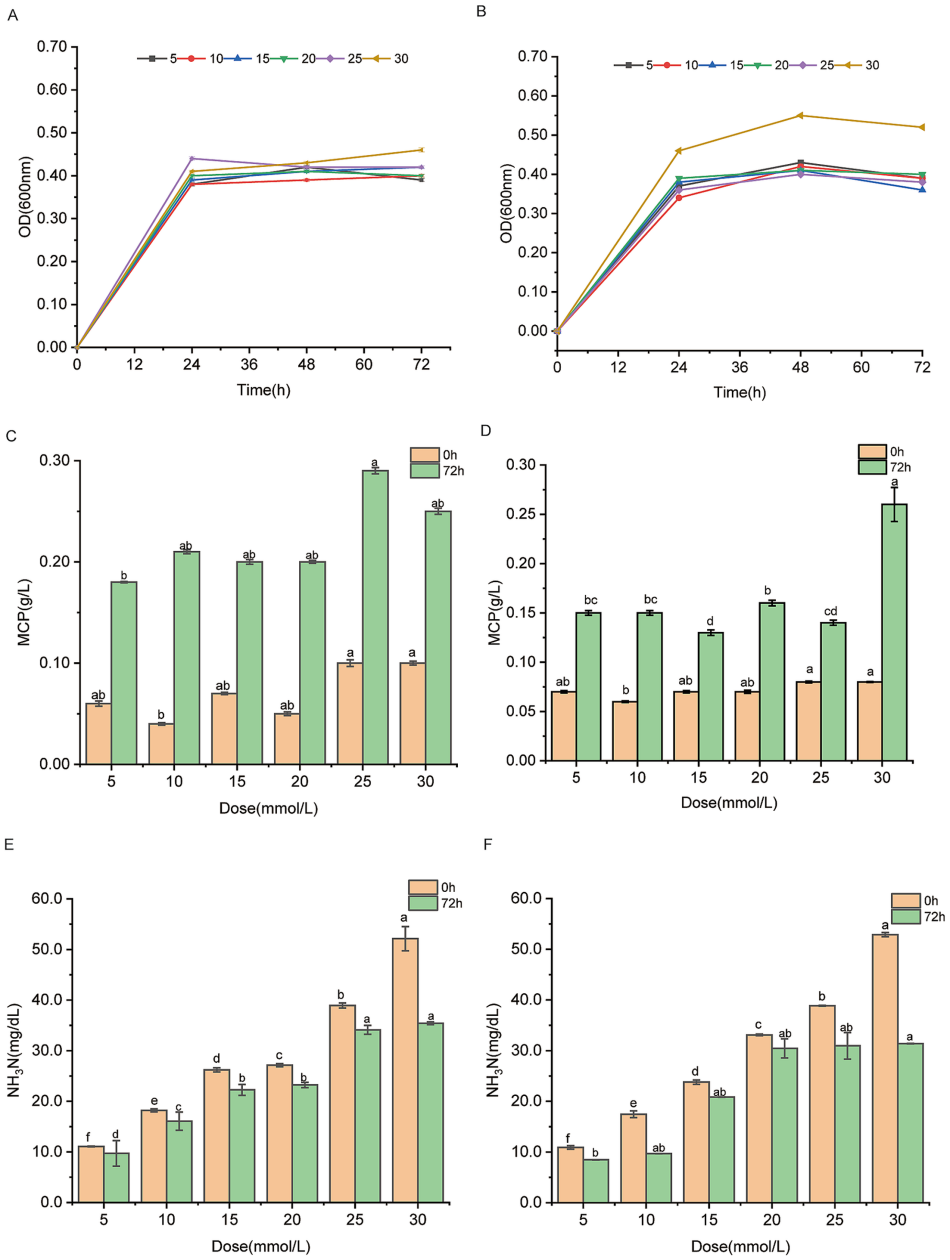
Ammonia nitrogen utilizing capacities of *Lactobacillus agilis* W70 and *Lactobacillus fermentum* M50 with 5, 10, 15, 20, 25, 30 mmol/L ammonium sulfate as the sole nitrogen source. **(A,B)** Growth curves over 72 h. **(C,D)** MCP yields at 0 and 72 h. **(E,F)** Ammonia nitrogen utilizing capacities at 0 and 72 h. Data are shown as mean ± standard deviation of three experiments. Different letters refer to statistically significant differences at *p* < 0.05.

Testing different ammonium sulfate concentrations helps assess the strains’ stability and adaptability under varying nitrogen source, ensuring that the selected strains exhibit consistent and reliable performance *in vitro* and *in vivo*. When ammonium sulfate was provided as the sole nitrogen sources at concentrations of 5, 10, 15, 20, 25, and 30 mmol/L, the W70 and M50 strains exhibited higher growth (OD_600nm_ level), indicating their tolerance to high levels of ammonium sulfate, which is a necessary prerequisite (Figures 2A,B). With the increase of ammonium sulfate concentration, the W70 and M50 strains displayed a trend of increased MCP. The ammonia nitrogen concentrations of 25 mmol/L and 30 mmol/L were optimal for the MCP production of the W70 and M50 strains, respectively, suggesting that the strain requires a certain nitrogen concentration to support effective utilization of ammonia nitrogen (Figures 2C,D). When 30 mmol/L of ammonium sulfate was used as the sole nitrogen source, the W70 strain showed the highest ammonia nitrogen utilization, with ammonium sulfate levels decreasing from 52.13 mg/dL at 0 h to 35.4 mg/dL at 72 h, a 32.09% reduction (Figure 2E). Similarly, strain M50 demonstrated a strong capacity for ammonia nitrogen utilization at 30 mmol/L of ammonium sulfate, with concentrations dropping from 52.88 mg/dL to 31.39 mg/dL over 72 h, resulting in a 40.64% reduction (Figure 2F). The ammonia consumption rates of W70 and M50 were 23.24 mg/L/h and 29.85 mg/L/h, respectively, with 30 mmol/L ammonium sulfate as the sole nitrogen source. Correspondingly, the bacterial growth rates were 0.10 h^−1^ and 0.10 h^−1^ under the same conditions.

The ability of the identified strains to degrade cellulose, starch, protein, and lipid was tested using cellulase, amylase, protease, and lipase activity plates. The results reveal that neither the W70 nor the W50 strain produced enzymes to degrade these substrates.

### 3.2 *In vitro* rumen fermentation

Facultative anaerobic *L. agilis* W70 and strictly anaerobic *L. fermentum* M50 were selected for the ruminal fermentation experiment. Supplementation of 1 × 10^9^ cfu/g of W70 and M50 had no effects on the pH, gas volume, and DMD (Table 2). In comparison with the CTL group, supplementation with the W70 strain significantly decreased the NH_3_N concentration (*p* < 0.01) and A:P ratio (*p* < 0.01), and increased the yields of total VFA (*p* < 0.05), acetate (*p* < 0.05), propionate (*p* < 0.05), isobutyrate (*p* < 0.01), butyrate (*p* < 0.01), isovalerate (*p* < 0.01), valerate (*p* < 0.01), MCP (*p* < 0.01), GDH (*p* < 0.05), and GS (*p* < 0.05). Supplementation with M50 strain increased acetate and MCP yields compared to the CTL group, without affecting NH_3_N levels. In combination with the screening and *in vitro* fermentation results, the W70 strain has more potential for NH_3_N utilization, total VFA/MCP production, and enzyme GDH/GS production. Therefore, this strain was characterized in further detail.

**Table 2:** Effect of *L. agilis* W70 and *L. fermentum* M50 on rumen fermentation *in vitro*^1^.

Fermentation characteristic	CTL	M50	W70	SEM	*p* value
pH	6.50	6.49	6.49	0.01	0.47
Gas (mL)	61.4	73.1	68.5	6.7	0.45
DMD (%)	49.49	44.00	44.33	2.59	0.31
Total VFA (mmol/L)	38.91^b^	47.42^b^	64.42^a^	3.00	0.01
Acetate (mmol/L)	27.02^b^	32.06^a^	42.16^a^	1.87	0.01
Propionate (mmol/L)	6.48^b^	8.19^b^	11.14^a^	0.57	0.01
Isobutyrate (mmol/L)	0.33^b^	0.45^b^	0.72^a^	0.04	<0.01
Butyrate (mmol/L)	4.05^b^	5.33^b^	8.18^a^	0.41	<0.01
Isovalerate (mmol/L)	0.61^b^	0.82^b^	1.33^a^	0.07	<0.01
Valerate (mmol/L)	0.43^b^	0.57^b^	0.89^a^	0.04	<0.01
A:P	4.23^a^	3.98^b^	3.82^b^	0.07	<0.01
NH_3_-N (mg/dL)	23.22^a^	21.63^ab^	19.46^b^	0.78	<0.01
MCP (g/L)	0.16^c^	0.19^b^	0.21^a^	0.01	<0.01
GDH (U/mL)	0.59^b^	0.62^ab^	0.74^a^	0.05	0.04
GS (U/mL)	0.02^b^	0.04^ab^	0.05^a^	0.01	0.04

1 Different letters a, ab, b of the same row refer to statistically significant differences withingroups (*p* < 0.05).

^2 CTL, control group; W70 = 1 × 109 cfu/g Ligilactobacillus agilis W70; M50 = 1 × 109 Limosilactobacillus fermentum cfu/g M50. 3 DMD, dry matter digestibility; Total VFA, total volatile fatty acid; A:P, the ratio of acetate and propionate; MCP, microbial protein; GDH, glutamate dehydrogenase; GS, glutamine synthetase.^

### 3.3 Phenotypic and genetic analysis of *Ligilactobacillus agilis* W70

**Figure 3:**
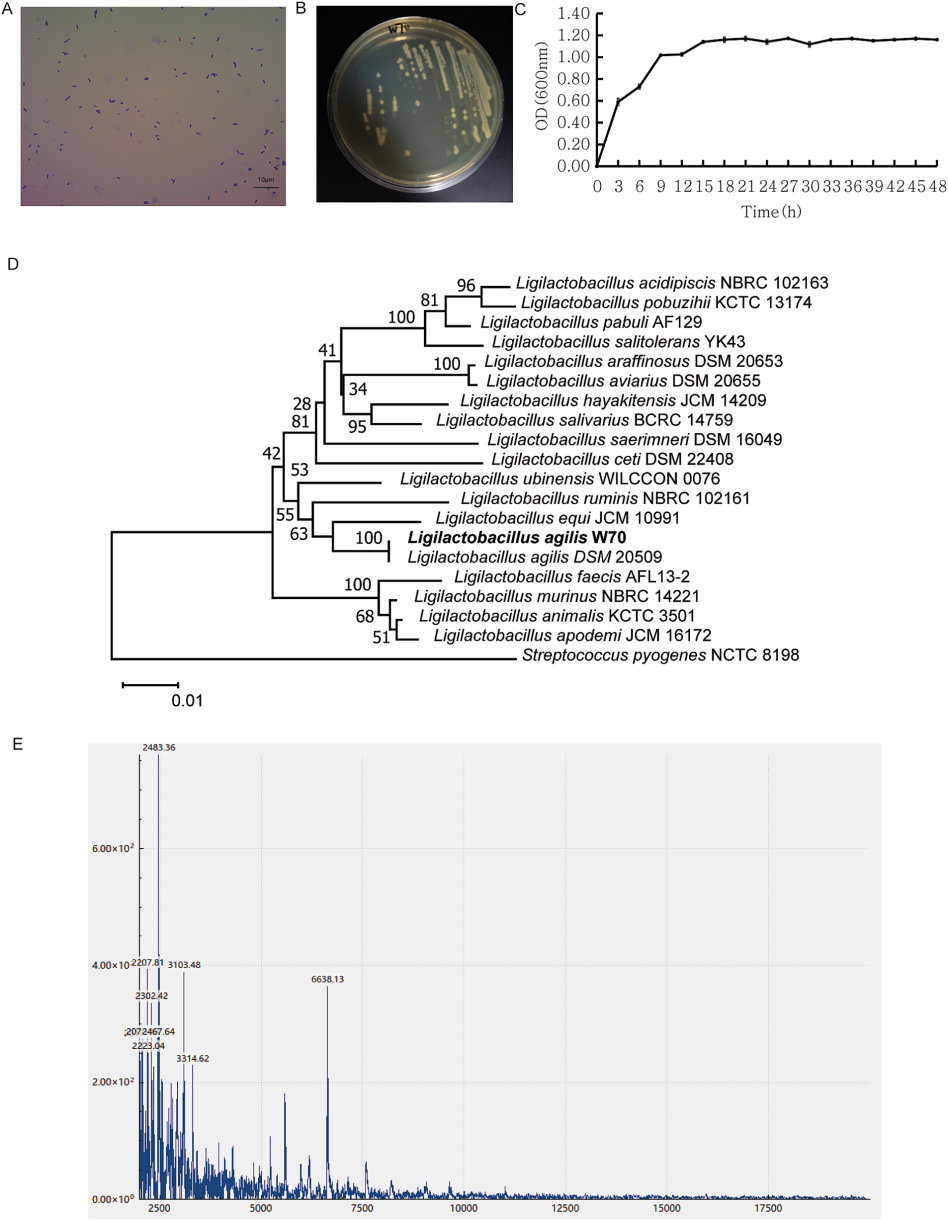
Morphological and physiochemical characters of *Ligilactobacillus agilis* W70. **(A)** Gram staining. **(B)** Colonies on MRS plate at 39°C. **(C)** Growth curve over 48 h in MRS broth at 39°C. Data are shown as mean ± standard deviation of triplicate. **(D)** Phylogenetic tree of the genus *Ligilactobacillus* constructed using the neighbor joining method based on the partial sequence of 16S rRNA gene. **(E)** MALDI-TOF MS profile of the ribosomal protein.

The W70 strain, identified as a gram-positive bacterium following gram staining, has rod-shaped cells measuring 0.14–0.57 μm × 1.03–1.90 μm (width × length) (Figures 3A,B). The cells were found singly or in pairs as shown by gram staining (Figure 3A). In the MRS liquid medium, the W70 strain cultured under facultative anaerobic condition at 39°C for 48 h showed an OD_600 nm_ maximum of 1.10 ± 0.01 after incubation for 15 h (Figure 3C). Based on the partial sequences of 16S rRNA gene and spectra of ribosomal proteins, the W70 strain was phylogenetically related to the genus *Ligilactobacillus* (Figures 3D,E).

**Figure 4:**
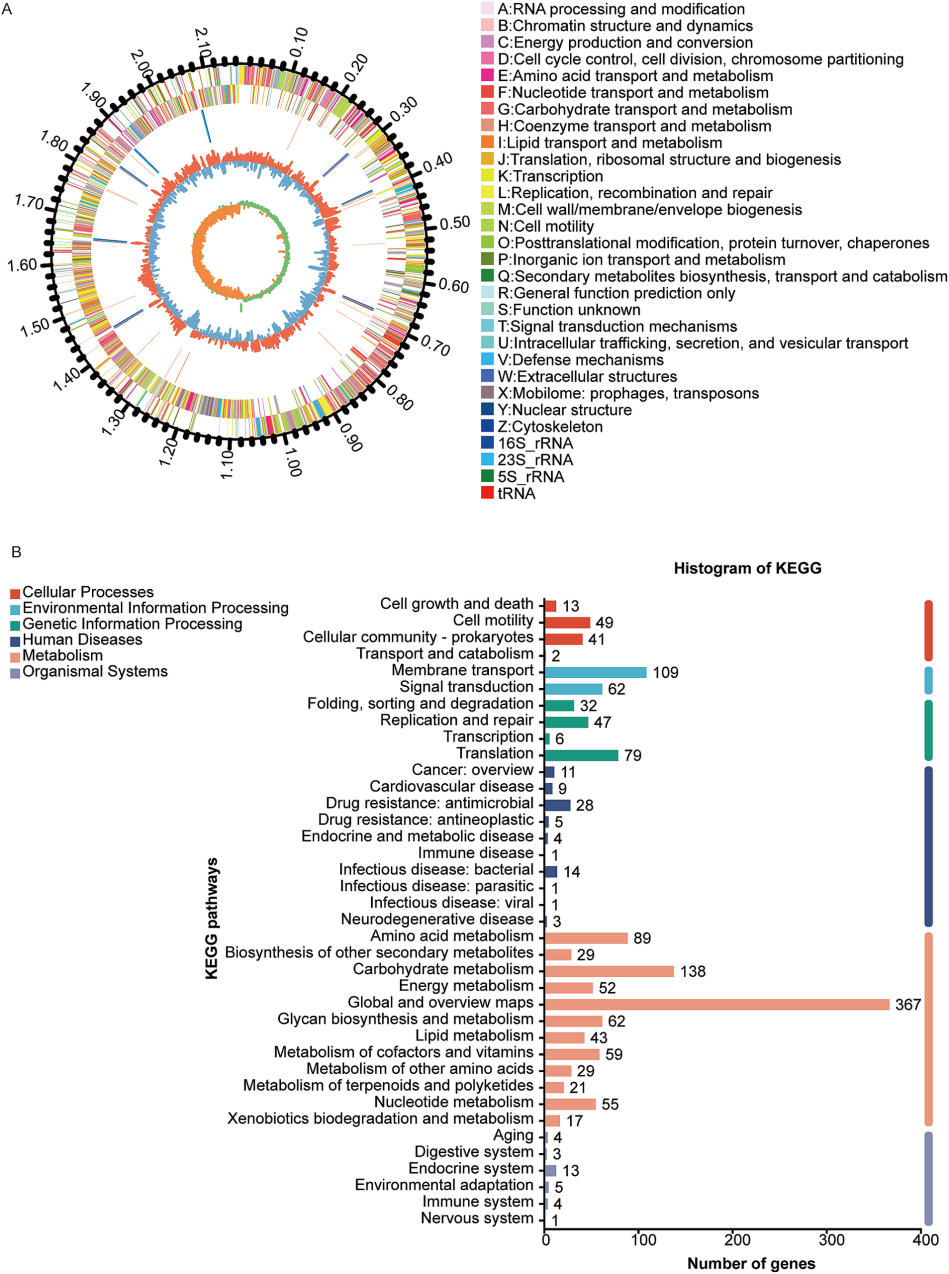
Genome analysis of *Ligilactobacillus agilis* W70. **(A)** Circle diagram of the nuclear genome. From the outside to the inside are the genome size, coding genes (sense), coding genes (antisense), tRNAs (red) and rRNAs (5S rRNA,16S rRNA and 23S rRNA,), prophage (purple), gene islands (yellow), insertion sequence (black), GC ratio and GC-skew. **(B)** KEGG classification of putative proteins.

The genome of the W70 strain is 2.17 Mb in size, with a G + C content of 41.7% (Figure 4A), and contains 2037 coding sequences, 90 tRNAs, 24 rRNA genes (8 copies of each 5S rRNA, 16S rRNA, and 23S rRNA), 32 sRNAs, 31 tandem repeats, 11 interspersed repeats, 11 gene islands, 1 prophage, 8 CRISPR repeats, 3 insertion sequences, and 12 transposons (Table 3). KEGG analysis indicated that most of the annotated genes are involved in carbohydrate metabolism (138 genes), membrane transport (109 genes), amino acid metabolism (89 genes), and metabolism of other amino acids (29 genes) (Figure 4B). GO analysis identified 834 genes related to molecular function (MF), 616 genes related to biological processes (BP), and 557 genes related to cellular components (CC) (Supplementary Figure S3A). COG analysis classified the putative proteins in three main groups, translation, ribosomal structure and biogenesis (193), carbohydrate transport and metabolism (172 genes), and amino acid transport and metabolism (144) (Supplementary Figure S3B).

**Table 3:** Statistics of the genomic structure of *L. agilis* W70.

Type	Number	Length (bp)	% genome
CDS	2,037	1,899,078	87.66
tRNA	90	6,709	0.35
16S rRNA	8	12,454	0.66
23S rRNA	8	23,302	1.23
5S rRNA	8	878	0.05
sRNA	32	5,160	0.24
Tandem repeat	31	14,821	0.78
Interspersed repeat	11	756	0.04
Genomic_island	11	188,397	9.92
Prophage	1	31,062	1.64
CRISPR	8	5,416	0.29
Insertion sequence	3	4,123	0.22
Transposon	12	9,504	0.50

The pathways of nitrogen metabolism in the W70 strain were predicted and presented in Supplementary Figure S4. The genes involved in ammonia assimilation, such as *glnA* and *gdhA*, were found to play key roles in the glutamate metabolism (Supplementary Table S2). The *gdhA* gene is 1,335 bp in length, encoding a deduced protein of 444 amino acids. Its similarity to sequences in the Swiss-Prot database is <62.67%, and < 62.90% in the PDB database. The homology model, based on the PDB template 5GUD, is presented in Table 4. As shown in Supplementary Figure S5 alignment with protein structure-resolved homologs of >50% identities, similar to other prokaryotic GDHs (5GUD and 5IJZ from *Corynebacterium glutamicum*, 4FCC and 3SBO from *Escherichia coli*, 3R3J from *Plasmodium falciparum* 3D7, and 1BGV, 1 K89, and 1AUP from *Clostridium symbiosum*), the *gdhA*-encoded protein consists of domain I (catalytic domain) that covers Met1-Ala204 and Asn369-Ile405, and domain II (nucleotide-binding domain) that covers Arg205-Ser368. Of them, Lys136, Ser 260, and Arg287 are predicted to be catalytic residues, and Arg94, Asp166, and Asn344 are putative binding sites to NADP^+^, respectively. The *glnA* gene is 1,344 bp in length, encoding a deduced protein of 447 amino acids. Its similarity to sequences in the Swiss-Prot database is <71.36%, and < 71.14% in the PDB database. The homology model, based on the PDB template 7TDP, is shown in Table 4. As shown in Supplementary Figure S6 alignment with protein structure-resolved homologs of >50% identities, similar to other prokaryotic GSs (7TDP from *Paenibacillus polymyxa*, 7TF6 from *Staphylococcus aureus*, 7TEN from *Listeria monocytogenes*, 4S0R from *Bacillus subtilis*, 8OOW from *Methermicoccus shengliensis* DSM 18856, 8UFJ from *Methanosarcina mazei* Go1, and 8OOL from *Methanothermococcus thermolithotrophicus*), the *glnA*-encoded protein is conserved in the catalytic domain and substrate binding site n. The expression of *gdhA* and *glnA* was significantly increased in modified MRS medium containing 30 mmol/L ammonium sulfate as the sole nitrogen source compared to 5 mmol/L ammonium sulfate (Supplementary Figure S7). These findings suggest that *gdhA* and *glnA* play a regulatory role in ammonia assimilation in W70.

**Table 4:** The *gdhA* and *glnA* deduced protein similarity and homology models.

Genes	ORF (bp)	Deduced proteins (aa)	Similarity to Swiss-prot (%)	Similarity to PDB (%)	PDB template	Homology models
*gdhA*	1,335	444	< 62.67	< 62.90	5GUD	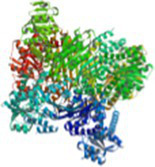
*glnA*	1,344	447	< 71.36	< 71.14	7TDP	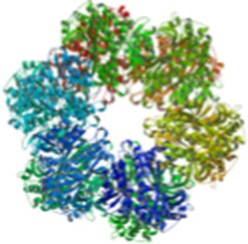

The pathways of biosynthesis of amino acids in the W70 strain were predicted and presented in Supplementary Figure S8. Genes involved in the biosynthesis of amino acids, such as proline, arginine, lysine, aspartate, asparagine, isoleucine, leucine, valine, methionine, serine, and histidine were also identified in the genome (Supplementary Table S3). The pathways of propionate metabolism in the W70 strain were predicted and presented in Supplementary Figure S9. The genes involved in propionate metabolism, such as *ldh, pflD, pta,* and *ackA*, were found to play key roles in the propionate production (Supplementary Table S4).

## 4 Discussion

Nitrogen is an essential macronutrient for microbial growth and basic metabolic processes (Chen et al., 2023). The degradation and utilization rate of nitrogen sources varies depending on the species and strains of ruminal bacteria (Cantalapiedra-Hijar et al., 2023). Some ruminal bacteria contribute to excess ammonia accumulation, resulting in a low yield of MCP (Qi et al., 2024). Therefore, identifying novel ammonia nitrogen utilizing bacteria and understanding their role in promoting nitrogen conversion in the rumen will provide knowledge to manipulate the functional microbes for potential application in the ruminant industry (Pfau et al., 2023).

### 4.1 *Ligilactobacillus agilis* W70 and *Limosilactobacillus fermentum* M50 as ammonia nitrogen utilizing strains

In the present study, 115 ruminal bacteria isolated from rumen fluid samples were tested in a medium with ammonium sulfate as the sole nitrogen source. The OD_600nm_ values were employed as indicators to identify the strain’s ammonia nitrogen utilizing capacity. Only the strains capable of utilizing ammonium sulfate were able to grow; those that could not utilize this nitrogen source did not survive. A previous study on ruminal ammonia nitrogen utilizing bacteria indicated that *Ruminococcus albus* 8 exhibits a preference for ammonia due to its higher growth rate (Kim et al., 2014). Among the tested strains, 10 strains which included *Bacillus safenisis* B55, *Lactobacillus agilis* W70, *Bacillus safenisis* B52, *Bacillus safenisis* F79, *Weissella confuse* W26, *Enterococcus faecium* F42, *Lactobacillus fermentum* M50, *Enterococcus lactis* M21, *Enterococcus faecium* B2, and *Lactobacillus salivarius* X36 had the highest OD_600nm_ levels in the presence of 30 mmol/L ammonium sulfate compared to others. Similarly, previous research has shown that certain probiotics, including *Bacillus* and lactic acid bacteria, can reduce NH_3_ emissions in rumen gas fermentation and animal studies (Sharma et al., 2017; Park et al., 2016; Mi et al., 2019) which agrees with the findings of the current study.

In this study, ammonium sulfate at 30 mmol/L was used for the secondary screening test of ammonia nitrogen utilization capacity. This concentration was selected based on findings by Liu et al. (2020), who reported that *Bacillus velezensis* LG37, isolated from an aquaculture pond, exhibited increased growth with rising ammonium sulfate concentrations (5, 10, 15, 20, 25, and 30 mmol/L), reaching maximum growth at 30 mmol/L. This screening allowed us to conduct an accurate evaluation of not only bacterial growth but also the strains’ ability to utilize ammonia nitrogen and produce metabolic products. As a result, *L. agilis* W70 and *L. fermentum* M50 decreased ammonia nitrogen and increased MCP to a greater extent than the four other tested strains. The optimal ammonia nitrogen concentrations for MCP production were identified as 25 mmol/L for *L. agilis* W70 and 30 mmol/L for *L. fermentum* M50 in this study, with excessive nitrogen concentrations not promoting further MCP production, consistent with findings by Zurak et al. (2023). This limitation may be due to metabolic overload or interference with other physiological functions of the strains (He et al., 2023). Previous research has shown that *Lactobacillus plantarum,* when added to pig manure, reduced NH_3_ emissions by 36% (Huang and Li, 2006), supporting our findings on the ammonia utilizing capacity of *Lactobacillus* in lowering ammonia levels, even in different contexts. In this study, *L. agilis* W70 and *L. fermentum* M50, which exhibited the highest ammonia nitrogen utilization capacity at 30 mmol/L ammonium sulfate, reduced ammonia nitrogen by 32.09 and 40.64%, respectively.

### 4.2 *Ligilactobacillus agilis* W70 reduced ammonia concentration and increased MCP production *in vitro* rumen fermentation

In order to make a direct comparison of ammonia nitrogen utilizing ability between the W70 and M50 strains, *in vitro* rumen fermentation was carried out. The *in vitro* fermentation replicates the rumen environment to provide a realistic assessment of the strain’s performance under conditions similar to those in the actual rumen, while also evaluating whether they have any negative impact on rumen function in a more complex and representative fermentation setting. The pH in this study was within the optimal rumen range of 6.0–7.0, as indicated by Wang et al. (2023). pH influences the activity of rumen microorganisms and the efficiency of rumen fermentation, as strain functionality is influenced by the complex rumen environment (Sanjorjo et al., 2023). Therefore, the lack of influence on pH is a positive finding in this case. In addition supplementation of 1 × 10^9^ cfu/mL of the W70 strain had no effect on the gas volume and DMD, emphasizing no negative influence on rumen fermentation. The W70 strain accelerated the utilization of NH_3_N in the rumen through MCP synthesis, thereby maximizing MCP production without wasting nitrogen in this study. MCP is synthesized by rumen microorganisms from NH_3_N, peptides, and amino acids in the rumen, which subsequently provides 50–90% rumen bypass protein for dairy cows (Li et al., 2023). Maintaining a proper NH_3_N balance is essential, as insufficient NH_3_N limits MCP production, while excess NH_3_N can result in nitrogen loss and waste (Zurak et al., 2023). The reduction in ammonia and increase in MCP is potentially associated with the higher expression levels of enzymes GDH and GS of strain W70 in the rumen. Although this mechanism needs to be further clarified as the enzymes were measured in the rumen fluid not the strains. VFAs are the primary energy pools in rumen and hindgut in ruminants for physiological activities, including growth, reproduction, and lactation (Sanz-Fernandez et al., 2020). The decrease in the A:P ratio of the W70 strain in the TMR diet indicated a shift toward more propionate production relative to acetate, which is beneficial for ruminants due to the difference in energy yield, improved feed efficiency, and reduced methane emissions (Russell, 2002). The production of propionate may be linked to the W70 strain itself, as its genome includes a KEGG carbohydrate metabolism pathway related to propionate synthesis from propionyl-CoA. The lower NH_3_N concentrations and A:P ratio and higher MCP indicated that the W70 strain contributed to nitrogen transformation and propionate production, without impairing effects *in vitro* rumen fermentation.

### 4.3 Genomic information of nitrogen metabolism of *Ligilactobacillus agilis* W70

Ammonia nitrogen utilizing bacteria typically use ammonia assimilation pathways to convert ammonia into glutamate and glutamine (Zhu et al., 2023), which are then involved in protein synthesis in the rumen. To examine the functionality of the W70 strain, a genome sequence analysis was conducted. KEGG analysis revealed that annotated genes of the W70 strain are involved in various metabolic processes which include: carbohydrate metabolism (138 genes), membrane transport (109 genes), amino acid metabolism (89 genes), and metabolism of other amino acids (29 genes). The W70 strain contains the genes *gdhA* and *glnA*, which are crucial for glutamate metabolism. Specifically, *gdhA* encodes GDH, responsible for utilizing ammonia to synthesize glutamate (De Mendonça Lopes et al., 2023), while *glnA* encodes GS, which converts glutamate to glutamine (Zhao et al., 2023). GDH and GS are well documented in ammonia assimilation processes (Wang et al., 2015), and are probably key to the enhanced ammonia utilization observed in the W70 strain. The assimilation of ruminal ammonia is typically mediated by GDH or GS (Nguyen et al., 2023). GDH is particularly effective when ammonia concentrations are high (Shilpha et al., 2023). Furthermore, *gdhA* and *glnA* have been identified as key genes of ammonia emission reduction in *Enterococcus faecium* strain C2 and *Bacillus coagulans* strain B1, reducing ammonia emission rates by 53.60 and 31.38%, respectively (Xiao et al., 2021). The high ammonia concentration used in this study (up to 30 mmol/L) might activate the GDH pathway in the W70 strain. As reported by Tomita et al. (2017), the GDH from *Corynebacterium glutamicum*, *Cg*GDH (PDB: 5GUD), showed the highest catalytic efficiency against NADPH. Thus the *gdhA e*ncoded protein with high identity to *Cg*GDH (62.90%) is inferred to own similar high catalytic efficiency and make great contribution to the ammonia utilization of strain W70. And as reported by Travis et al. (2022), the deduced protein of *glnA* forms a ligand with Met-Sox-P in the presence of ATP. In the complexes, the Glu307-Asp55 interaction would shield the Met-Sox-P from attack by bulk solvent as well as facilitate proton abstraction of the ammonium to form glutamine. These findings imply that *glnA* may encode highly efficient enzymes for ammonia utilization in strain W70.

In addition to *gdhA* and *glnA*, W70’s genome also includes genes involved in the biosynthesis of various amino acids such as proline, arginine, lysine, aspartate, asparagine, isoleucine, leucine, valine, methionine, serine, and histidine. Genes related to nitrogen metabolism are advantageous for further applications in the ruminant industry to enhance nitrogen utilization efficiency (Zhang et al., 2023). Similarly, genes involved in ammonia assimilation and the biosynthesis of alanine, aspartate, glutamate, and lysine were found in *Succinivibrio dextrinosolvens* strain Z6, which showed maximum growth in ammonia containing medium (Hailemaria et al., 2020). Moreover, supplementing dairy cows with 15 g/day of *Bacillus megaterium* BM1259 (1 × 10^8^ cfu/g) improved nitrogen utilization and milk production, which was closely related to the nitrate assimilation-related genes and pathways suggesting this strain may be advantageous when fed *in vivo* (Deng et al., 2021). Therefore, the *gdhA* and *glnA* genes in the W70 strain, along with genes involved in amino acid biosynthesis, contribute to its effective ammonia nitrogen utilization capability *in vitro* and suggest potential applications for improving nitrogen utilization efficiency *in vivo*.

## 5 Conclusion

In summary, *Lactobacillus agilis* W70 and *Lactobacillus fermentum* M50 isolated from rumen fluid were found to have greater ammonia nitrogen utilizing capabilities than other tested strains. Among them, the W70 strain showed higher efficiency in NH_3_N utilization and MCP synthesis *in vitro* rumen fermentation. Genome analysis revealed that *gdhA* and *glnA* coding for putative glutamate dehydrogenase and glutamine synthetase, respectively, are potentially key genes of the W70 strain in regulating nitrogen metabolism, as demonstrated in tube tests and *in vitro* experiments. Further studies on application of W70 strain in feed will be conducted to improve the nitrogen utilization efficiency in the ruminant industry.

## Data Availability

The datasets presented in this article are not readily available because the relevant strains are patented. Requests to access the datasets should be directed to the corresponding author.

## Footnotes

^1^ http://www.megasoftware.net/

^2^ https://github.com/tseemann/barrnap

^3^ https://www.uniprot.org/blast

^4^ http://www.rcsb.org

^5^ https://swissmodel.expasy.org/interactive

^6^ https://espript.ibcp.fr/ESPript/cgi-bin/ESPript.cgi

^7^ https://www.ncbi.nlm.nih.gov/tools/primer-blast
