# Supplementary material for: Ligilactobacillus agilis W70: a probiotic with capacity to enhance ammonia assimilation in the rumen
Source: Front Microbiol. 2025 Jan 17;15:1498109. doi: 10.3389/fmicb.2024.1498109 (PMC11782116; doi:10.3389/fmicb.2024.1498109)
Supplement: Supplementary file 1 [file Data_Sheet_1.pdf]

Supplementary materials

No.	Species	Strain	5mmol/L	10mmol/L	15mmol/L	20mmol/L	25mmol/L	30mmol/L
1	*Ligilactobacillus agilis*	X62	0.23	0.23	0.23	0.24	0.32	0.32
2	*Bacillus safensis*	F60	0.28	0.28	0.28	0.28	0.37	0.38
3	*Bacillus subtilis*	W4	0.29	0.29	0.30	0.28	0.37	0.36
4	*Bacillus safensis*	W42	0.29	0.29	0.33	0.32	0.38	0.41
5	*Bacillus safensis*	W56	0.28	0.28	0.29	0.35	0.40	0.36
6	*Limosilactobacillus fermentum*	B32	0.29	0.29	0.31	0.34	0.45	0.43
7	*Limosilactobacillus fermentum*	M21	0.26	0.26	0.26	0.27	0.25	0.29
8	*Ligilactobacillus agilis*	X19	0.35	0.35	0.30	0.33	0.31	0.24
9	*Ligilactobacillus agilis*	X64	0.27	0.27	0.28	0.29	0.41	0.44
10	*Bacillus safensis*	F79	0.31	0.31	0.32	0.35	0.50	0.48
11	*Ligilactobacillus agilis*	W43	0.29	0.29	0.31	0.41	0.47	0.46
12	*Ligilactobacillus agilis*	W64	0.28	0.28	0.29	0.42	0.45	0.46
13	*Bacillus safensis*	B52	0.30	0.30	0.30	0.40	0.49	0.50
14	*Bacillus safensis*	M42	0.31	0.31	0.30	0.27	0.23	0.44
15	*Ligilactobacillus agilis*	X44	0.27	0.27	0.24	0.27	0.24	0.22
16	*Enterococcus camelliae*	S35	0.26	0.26	0.27	0.28	0.42	0.43
17	*Weissella confusa*	W26	0.32	0.32	0.31	0.38	0.47	0.48
18	*Limosilactobacillus fermentum*	W45	0.29	0.29	0.30	0.40	0.46	0.47
19	*Ligilactobacillus agilis*	W70	0.30	0.30	0.31	0.45	0.48	0.50
20	*Bacillus safensis*	B55	0.30	0.30	0.29	0.31	0.47	0.51
21	*Enterococcus lactis*	M45	0.25	0.25	0.26	0.27	0.26	0.40
22	*Ligilactobacillus agilis*	X46	0.24	0.24	0.24	0.24	0.22	0.19
23	*Bacillus safensis*	F19	0.25	0.25	0.25	0.27	0.24	0.24
24	*Ligilactobacillus agilis*	W40	0.29	0.29	0.29	0.32	0.29	0.33
25	*Ligilactobacillus agilis*	W52	0.30	0.30	0.29	0.32	0.26	0.33
26	*Limosilactobacillus fermentum*	W72	0.29	0.29	0.28	0.33	0.26	0.33

Figure S1. Heat map of 26 strains growth capacity (OD_{600nm}) under facultative anaerobic gas condition with ammonium sulfate (5, 10, 15, 20, 25, and 30 mmol/L) as the sole nitrogen source.

No.	Species	Strain	5mmol/L	10mmol/L	15mmol/L	20mmol/L	25mmol/L	30mmol/L
1	*Enterococcus lactis*	X1	0.24	0.26	0.28	0.55	0.52	0.57
2	*Enterococcus faecium*	Y34	0.23	0.24	0.28	0.27	0.28	0.27
3	*Ligilactobacillus salivarius*	P17	0.24	0.24	0.28	0.27	0.28	0.27
4	*Ligilactobacillus agilis*	P53	0.26	0.26	0.30	0.39	0.28	0.43
5	*Ligilactobacillus agilis*	S14	0.32	0.26	0.29	0.28	0.31	0.29
6	*Bacillus piscis*	B11	0.26	0.24	0.30	0.46	0.47	0.45
7	*Ligilactobacillus salivarius*	W14	0.32	0.35	0.37	0.36	0.37	0.36
8	*Ligilactobacillus salivarius*	F15	0.20	0.22	0.24	0.25	0.25	0.24
9	*Bacillus licheniformis*	F37	0.27	0.35	0.34	0.35	0.44	0.44
10	*Limosilactobacillus fermentum*	M18	0.25	0.35	0.34	0.36	0.31	0.34
11	*Limosilactobacillus fermentum*	X18	0.27	0.34	0.35	0.56	0.51	0.51
12	*Enterococcus lactis*	Y35	0.32	0.24	0.27	0.28	0.28	0.28
13	*Ligilactobacillus agilis*	P18	0.23	0.21	0.18	0.27	0.20	0.20
14	*Limosilactobacillus fermentum*	P74	0.26	0.27	0.37	0.38	0.37	0.36
15	*Limosilactobacillus fermentum*	S15	0.27	0.27	0.32	0.63	0.63	0.60
16	*Ligilactobacillus salivarius*	B12	0.28	0.28	0.30	0.50	0.47	0.46
17	*Limosilactobacillus fermentum*	W16	0.31	0.24	0.26	0.26	0.27	0.27
18	*Limosilactobacillus mucosae*	F18	0.24	0.24	0.27	0.33	0.30	0.29
19	*Ligilactobacillus agilis*	F41	0.26	0.39	0.37	0.38	0.38	0.40
20	*Enterococcus lactis*	M21	0.26	0.30	0.30	0.65	0.65	0.65
21	*Ligilactobacillus agilis*	X20	0.27	0.27	0.32	0.62	0.54	0.56
22	*Limosilactobacillus fermentum*	Y36	0.28	0.24	0.24	0.24	0.24	0.25
23	*Ligilactobacillus salivarius*	P19	0.34	0.32	0.31	0.28	0.30	0.28
24	*Ligilactobacillus agilis*	P77	0.27	0.27	0.32	0.44	0.44	0.45
25	*Enterococcus faecium*	S16	0.26	0.30	0.37	0.39	0.40	0.39
26	*Enterococcus faecium*	B13	0.26	0.30	0.33	0.59	0.54	0.53
27	*Ligilactobacillus agilis*	W18	0.27	0.27	0.31	0.32	0.33	0.31
28	*Limosilactobacillus fermentum*	F20	0.25	0.26	0.28	0.29	0.29	0.31
29	*Enterococcus faecium*	F42	0.27	0.26	0.32	0.67	0.67	0.66
30	*Limosilactobacillus fermentum*	M27	0.29	0.30	0.31	0.31	0.32	0.30
31	*Ligilactobacillus salivarius*	X36	0.26	0.27	0.32	0.74	0.63	0.64
32	*Limosilactobacillus fermentum*	Y45	0.33	0.30	0.31	0.29	0.28	0.28
33	*Ligilactobacillus agilis*	P32	0.34	0.32	0.33	0.29	0.29	0.27
34	*Enterococcus faecium*	P80	0.32	0.27	0.25	0.26	0.26	0.25
35	*Ligilactobacillus salivarius*	S17	0.26	0.27	0.39	0.43	0.44	0.39
36	*Ligilactobacillus salivarius*	B14	0.27	0.26	0.32	0.61	0.59	0.61
37	*Ligilactobacillus agilis*	W23	0.20	0.35	0.31	0.33	0.33	0.32
38	*Enterococcus faecium*	F21	0.27	0.28	0.30	0.31	0.31	0.32
39	*Ligilactobacillus salivarius*	F43	0.27	0.35	0.35	0.34	0.35	0.37
40	*Limosilactobacillus fermentum*	M43	0.29	0.30	0.33	0.31	0.32	0.33
41	*Enterococcus lactis*	X55	0.32	0.26	0.18	0.20	0.19	0.27
42	*Enterococcus lactis*	Y48	0.32	0.28	0.29	0.30	0.27	0.26
43	*Ligilactobacillus agilis*	P33	0.27	0.27	0.32	0.54	0.56	0.53
44	*Enterococcus lactis*	R13	0.32	0.30	0.30	0.29	0.28	0.27
45	*Lacticaseibacillus paracasei*	S18	0.27	0.27	0.30	0.66	0.63	0.62
46	*Limosilactobacillus fermentum*	B21	0.36	0.33	0.31	0.33	0.32	0.30
47	*Ligilactobacillus agilis*	F24	0.26	0.27	0.31	0.62	0.59	0.59
48	*Limosilactobacillus fermentum*	F44	0.27	0.29	0.31	0.39	0.45	0.45
49	*Limosilactobacillus fermentum*	M50	0.27	0.30	0.34	0.72	0.70	0.66
50	*Enterococcus lactis*	Y7	0.22	0.22	0.26	0.25	0.31	0.25
51	*Enterococcus lactis*	Y50	0.25	0.22	0.21	0.21	0.21	0.21
52	*Ligilactobacillus agilis*	P38	0.27	0.30	0.34	0.53	0.51	0.51
53	*Ligilactobacillus salivarius*	S3	0.27	0.27	0.36	0.38	0.37	0.35
54	*Enterococcus faecium*	B1	0.26	0.30	0.38	0.38	0.36	0.34
55	*Ligilactobacillus agilis*	B23	0.26	0.29	0.31	0.32	0.32	0.30
56	*Enterococcus faecium*	W37	0.27	0.30	0.33	0.33	0.34	0.34
57	*Limosilactobacillus mucosae*	F27	0.27	0.30	0.31	0.33	0.32	0.34
58	*Limosilactobacillus fermentum*	F48	0.37	0.27	0.23	0.23	0.56	0.24
59	*Limosilactobacillus fermentum*	M52	0.33	0.33	0.32	0.34	0.45	0.36
60	*Ligilactobacillus salivarius*	Y24	0.14	0.17	0.12	0.15	0.20	0.11

61	*Limosilactobacillus fermentum*	Y67	0.30	0.27	0.26	0.25	0.25	0.27
62	*Limosilactobacillus fermentum*	P39	0.27	0.27	0.33	0.57	0.56	0.55
63	*Limosilactobacillus fermentum*	S5	0.28	0.30	0.31	0.28	0.38	0.27
64	*Enterococcus faecium*	B2	0.27	0.29	0.33	0.70	0.62	0.64
65	*Ligilactobacillus agilis*	B24	0.26	0.33	0.34	0.31	0.30	0.28
66	*Limosilactobacillus fermentum*	F3	0.28	0.29	0.32	0.54	0.35	0.51
67	*Limosilactobacillus mucosae*	F30	0.27	0.29	0.32	0.56	0.56	0.55
68	*Enterococcus faecium*	F63	0.26	0.27	0.31	0.38	0.45	0.38
69	*Limosilactobacillus fermentum*	M55	0.27	0.29	0.30	0.43	0.37	0.41
70	*Limosilactobacillus fermentum*	Y26	0.18	0.19	0.20	0.20	0.23	0.21
71	*Limosilactobacillus fermentum*	P1	0.32	0.29	0.29	0.29	0.29	0.27
72	*Ligilactobacillus agilis*	P43	0.27	0.29	0.30	0.60	0.56	0.56
73	*Limosilactobacillus fermentum*	B8	0.33	0.30	0.28	0.29	0.27	0.26
74	*Limosilactobacillus fermentum*	W6	0.31	0.29	0.26	0.27	0.26	0.26
75	*Limosilactobacillus fermentum*	F9	0.37	0.33	0.30	0.32	0.32	0.32
76	*Limosilactobacillus fermentum*	F33	0.29	0.30	0.32	0.33	0.31	0.36
77	*Ligilactobacillus salivarius*	F74	0.31	0.27	0.31	0.29	0.27	0.27
78	*Limosilactobacillus fermentum*	M61	0.27	0.29	0.21	0.20	0.27	0.28
79	*Limosilactobacillus fermentum*	Y27	0.26	0.27	0.30	0.48	0.49	0.47
80	*Limosilactobacillus fermentum*	P2	0.26	0.20	0.27	0.27	0.27	0.29
81	*Limosilactobacillus fermentum*	P52	0.28	0.29	0.29	0.52	0.53	0.49
82	*Limosilactobacillus fermentum*	S13	0.26	0.29	0.27	0.53	0.54	0.46
83	*Limosilactobacillus fermentum*	B10	0.27	0.30	0.29	0.55	0.52	0.52
84	*Limosilactobacillus fermentum*	W12	0.26	0.29	0.30	0.46	0.45	0.40
85	*Limosilactobacillus fermentum*	F14	0.27	0.30	0.27	0.30	0.27	0.30
86	*Bacillus licheniformis*	F36	0.30	0.33	0.33	0.36	0.31	0.35
87	*Limosilactobacillus fermentum*	M15	0.36	0.34	0.34	0.35	0.33	0.34
88	*Limosilactobacillus fermentum*	M69	0.28	0.29	0.33	0.27	0.28	0.29
89	*Pediococcus acidilactici*	M78	0.21	0.29	0.22	0.21	0.24	0.22

Figure S2. Heat map of 89 strains growth capacity (OD_{600nm}) under anaerobic gas condition with ammonium sulfate (5, 10, 15, 20, 25, and 30 mmol/L) as the sole nitrogen source.

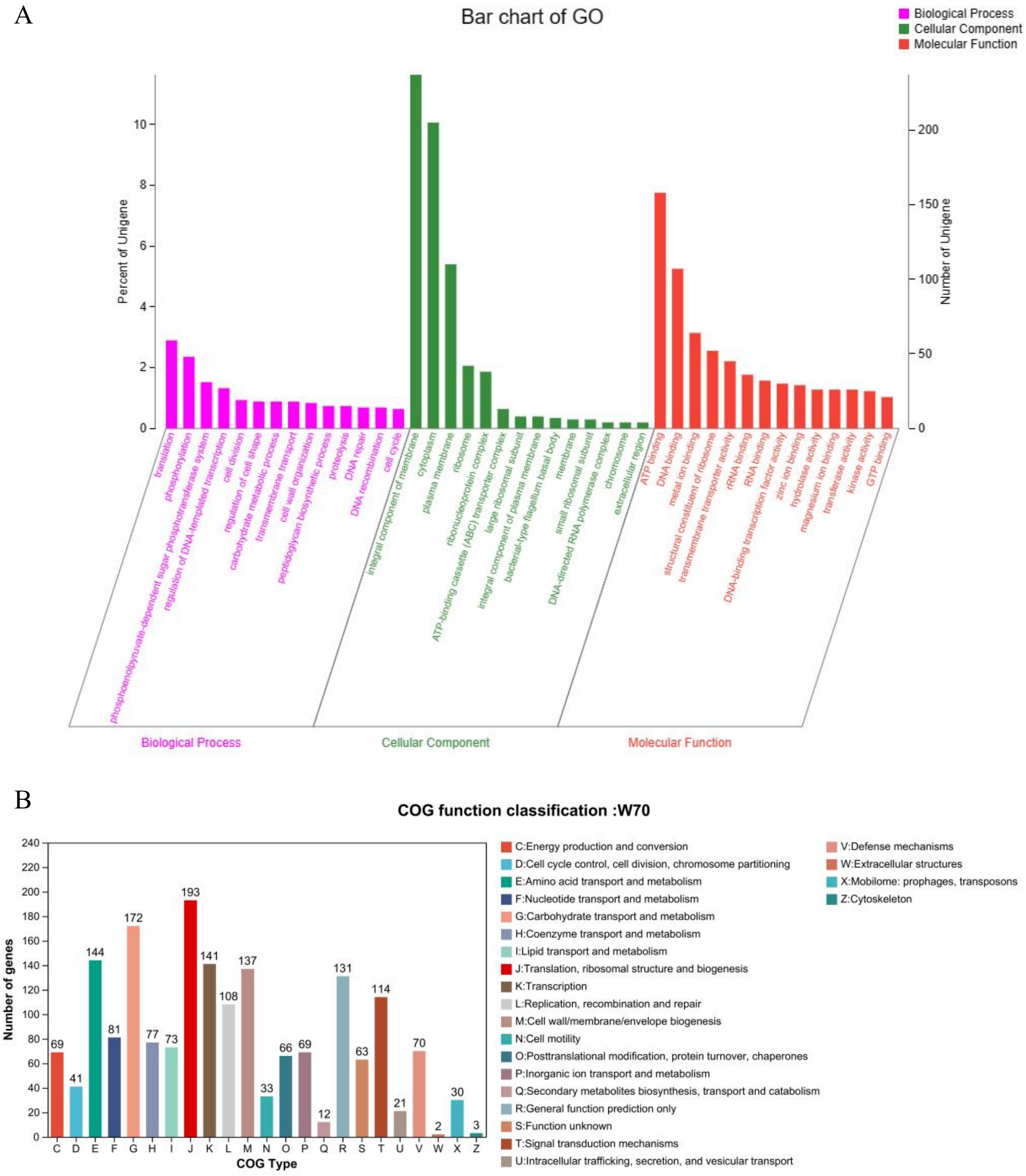

Figure S3. GO and COG annotation analysis of *Ligilactobacillus agilis* W70. **(A)** GO classification of putative proteins. **(B)** COG classification of putative proteins.

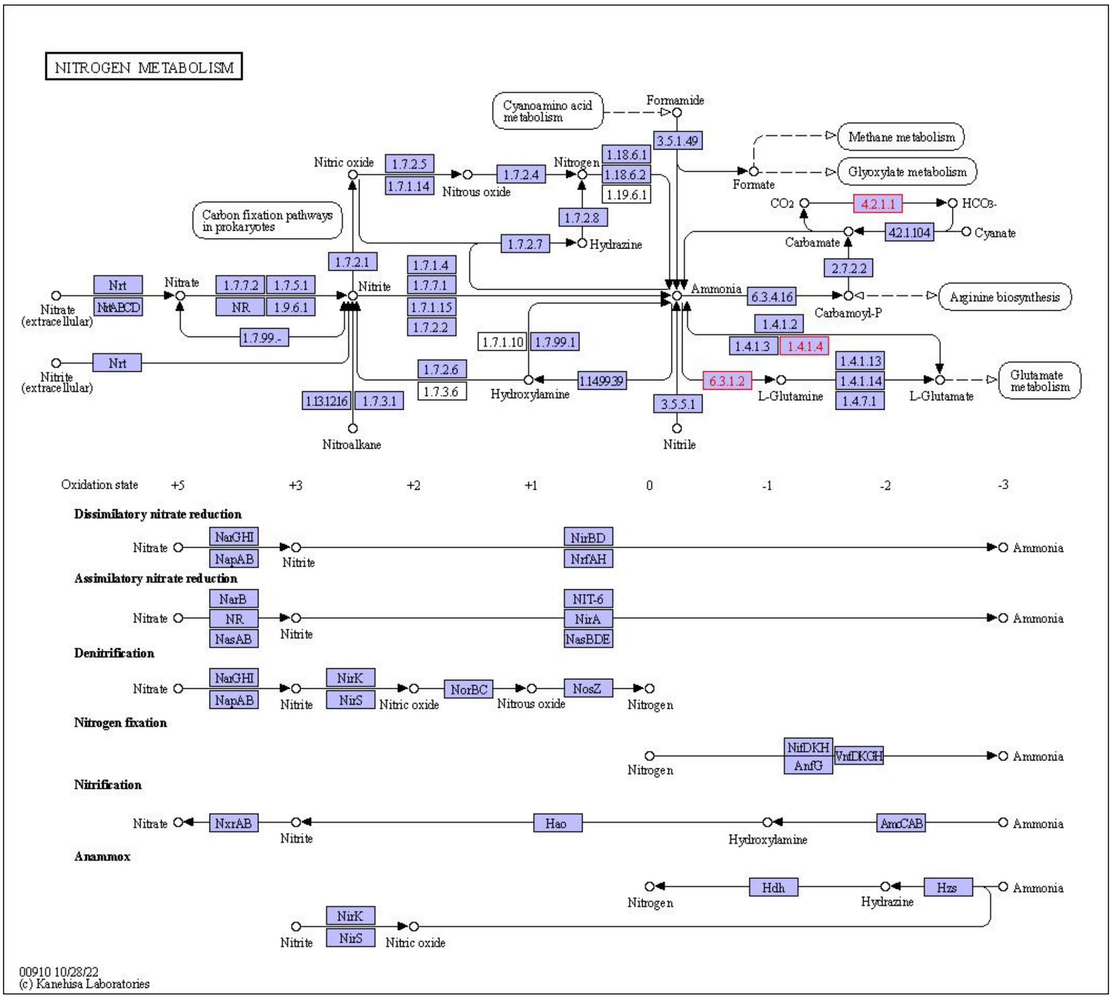

Figure S4. Nitrogen metabolism in *Ligilactobacillus agilis* W70 using the KEGG database. The red boxes represent the enzymes involved in nitrogen metabolism in strain W70. EC:6.3.1.2, glutamine synthetase; EC:1.4.1.4, glutamate dehydrogenase ($NADP^+$).

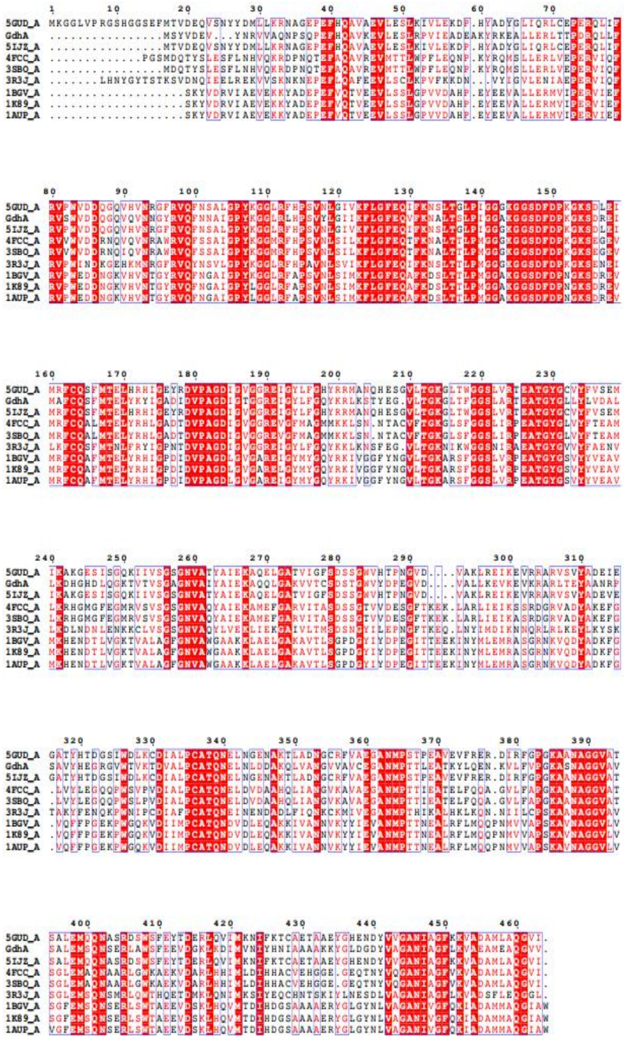

Figure S5. Multiple sequence alignment of the 5GUD, gdhA, 5IJZ, 4FCC, 3SBO, 3R3J, 1BGV, 1K89, and 1AUP proteins. (PDB templates 5GUD and 5IJZ from *Corynebacterium glutamicum*, 4FCC and 3SBO from *Escherichia coli*, 3R3J from *Plasmodium falciparum* 3D7, 1BGV, 1K89, and 1AUP from *Clostridium symbiosum,* and gdhA from *Ligilactobacillus agilis* W70)

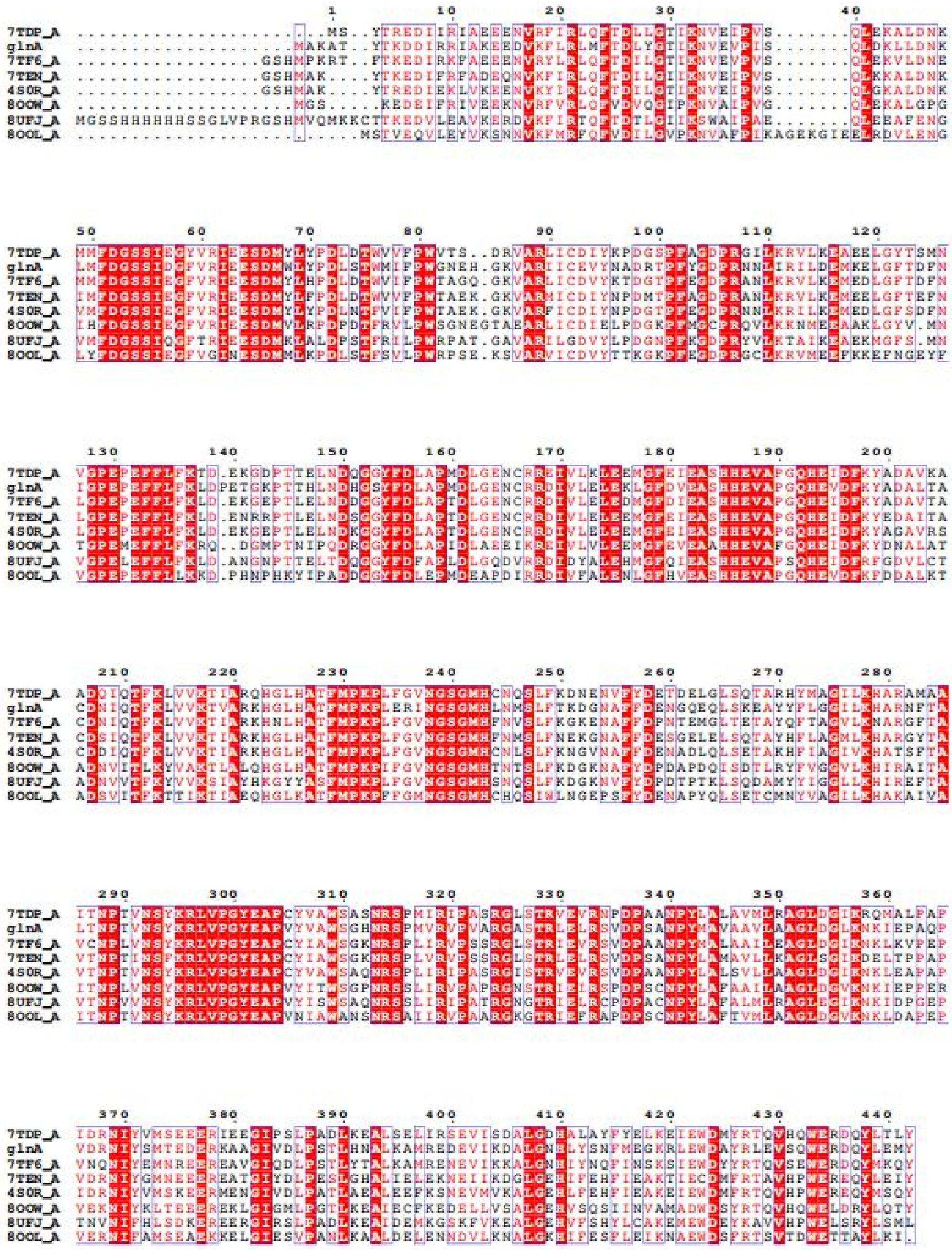

Figure S6. Multiple sequence alignment of the 7TDP, glnA, 7TF6, 7TEN, 4S0R, 8OOW, 8UFJ, and 8OOL proteins. (PDB templates 7TDP from *Paenibacillus polymyxa*, 7TF6 from *Staphylococcus aureus*, 7TEN from *Listeria monocytogenes*, 4S0R from *Bacillus subtilis*, 8OOW from *Methermicoccus shengliensis* DSM 18856, 8UFJ from *Methanosarcina mazei* Go1, and 8OOL from *Methanothermococcus thermolithotrophicus,* and glnA from *Ligilactobacillus agilis* W70)

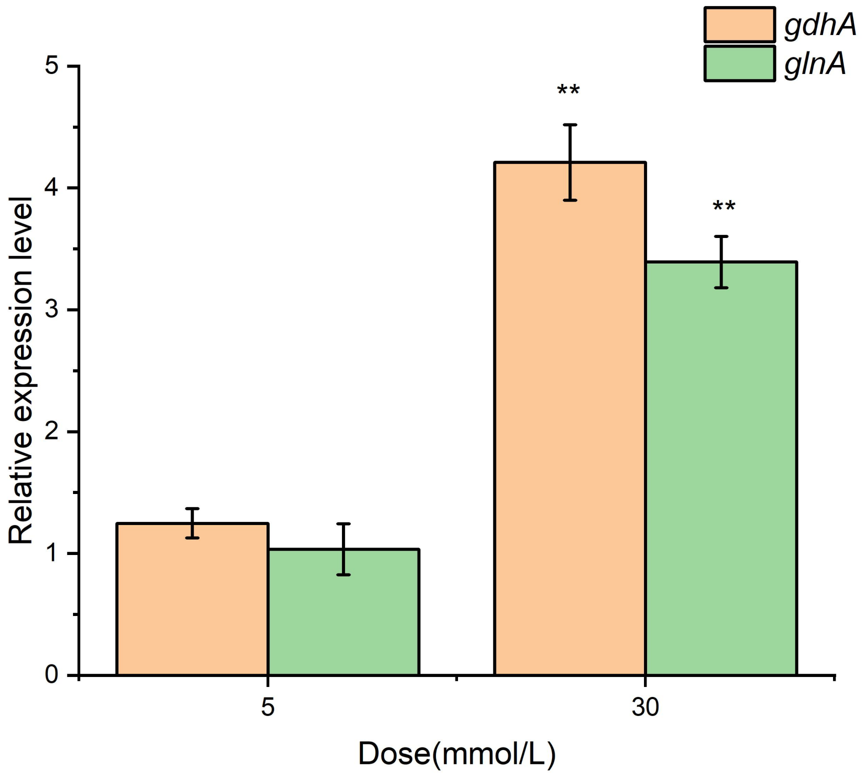

Figure S7. Relative expression of *gdhA* and *glnA* in 5 mmol/L and 30 mmol/L ammonium sulfate as the sole nitrogen source modified MRS medium. These results are means ± SD. **P* < 0.05, ***P* < 0.01 versus control.

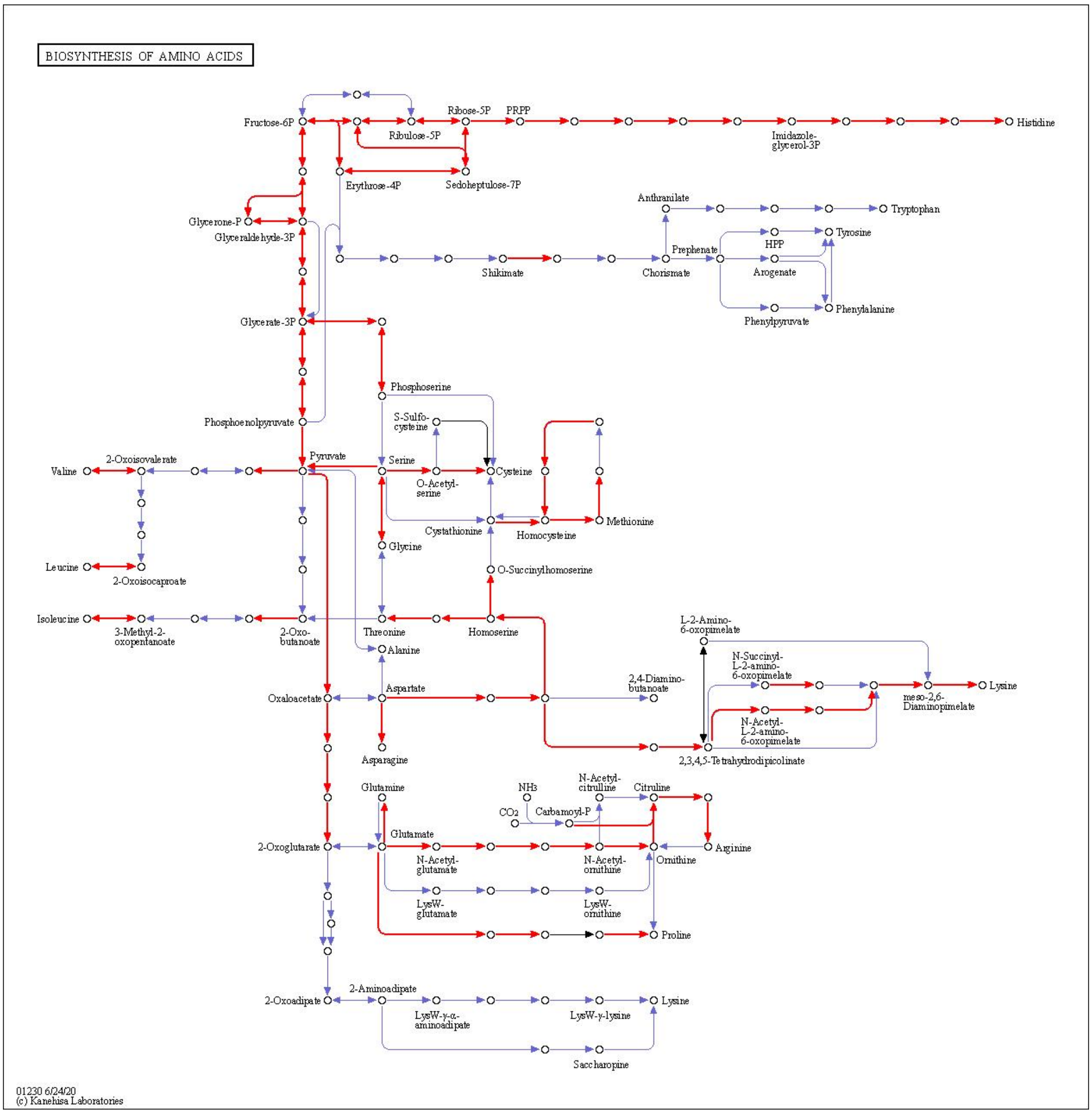

Figure S8. Biosynthesis pathway of amino acids in *Ligilactobacillus agilis* W70 using the KEGG database.

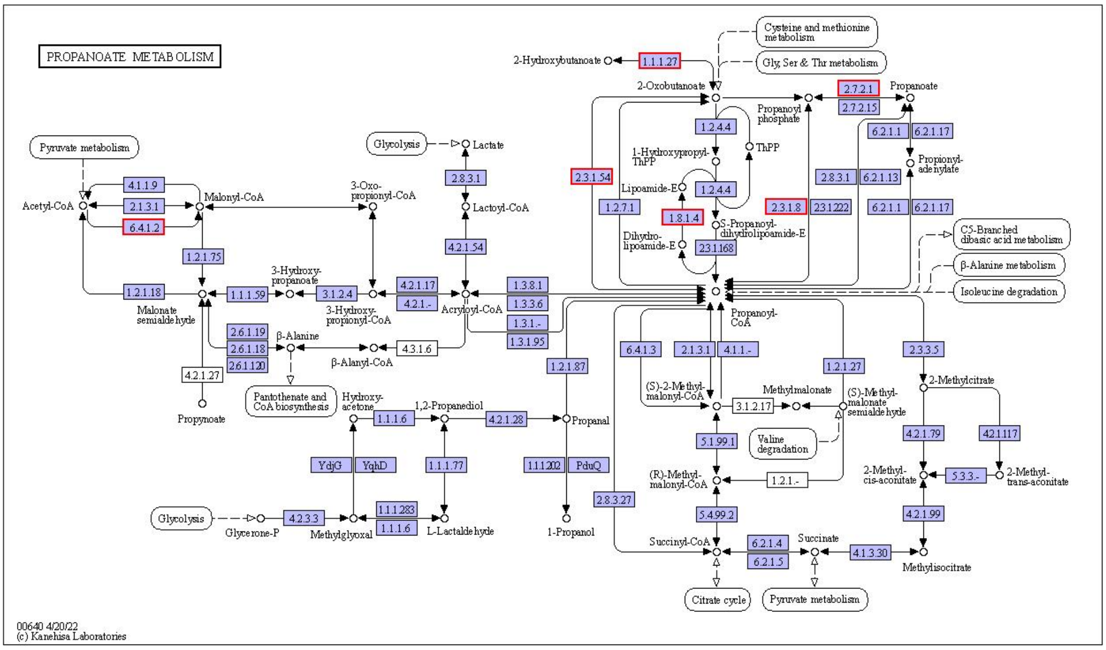

Figure S9. Propionate metabolism in *Ligilactobacillus agilis* W70 using the KEGG database. The red boxes represent the enzymes involved in propionate metabolism in strain W70. EC:1.1.1.27, L-lactate dehydrogenase; EC:2.3.1.54, formate C-acetyltransferase; EC:2.3.1.8, phosphate acetyltransferase; EC:2.7.2.1, acetate kinase.

Table S1. Primers of *gdhA* and *glnA* in RT-qPCR analysis

Target gene	Forward primer (5’ to 3’)	Reverse primer (5’ to 3’)	Product size (bp)
16S rDNA	ACTCCTACGGGAGGCAGCAG	ATTACCGCGGCTGCTGGC	180
gdhA	CTTACAAGGGCGGCTTACGA	CGCCGATTGGAAGGGAAGTA	108
glnA	AGCAACGTAAACAGGGGCTT	ACGGCCCTAACTAACCCAAC	75

Table S2. The genes involved in ammonia assimilation of *Ligilactobacillus agilis* W70

Gene ID	gene1600	gene1769
Gene Name	*glnA*	*gdhA*
ChromosomeLocation	1577318-1578661	1753698-1755032
Description	type I glutamate--ammonia ligase	NADP-specific glutamate dehydrogenase
NR[1]	type I glutamate--ammonia ligase	NADP-specific glutamate dehydrogenase
Swiss-Prot	Glutamine synthetase OS=*Staphylococcus aureus* (strain MW2)	NADP-specific glutamate dehydrogenase OS=*Corynebacterium glutamicum* (strain ATCC 13032 / DSM 20300 / BCRC 11384 / JCM 1318 / LMG 3730 / NCIMB 10025)
KEGG[2]	K01915 (glutamine synthetase [EC:6.3.1.2])	K00262 (glutamate dehydrogenase $NADP^+$) [EC:1.4.1.4])
COG[3]	COG0174 (Glutamine synthetase)	COG0334 (Glutamate dehydrogenase/leucine dehydrogenase)
GO[4]	GO:0006542;GO:0005524;GO:0004356 (glutamine biosynthetic process; ATP binding; glutamate-ammonia ligase activity)	GO:0006520;GO:0000166;GO:0004352 (cellular amino acid metabolic process; nucleotide binding; glutamate dehydrogenase NAD^+activity)
Pfam	PF00120-Gln-synt_C;PF03951-Gln-synt_N (Glutamine synthetase, catalytic domain; Glutamine synthetase, beta-Grasp domain)	PF00208-ELFV_dehydrog; PF02812-ELFV_dehydrog_N (Glutamate/Leucine/Phenylalanine/Valine dehydrogenase; Glu/Leu/Phe/Val dehydrogenase, dimerisation domain)

[1]NR: Non-Redundant Protein Database;
[2]KEGG: Kyoto Encyclopedia of Genes and Genomes;
[3]COG: Clusters of Orthologous Groups of proteins; [4]GO: Gene Ontology

Table S3. The genes involved in amino acid biosynthesis of *Ligilactobacillus agilis* W70

Gene ID	KO ID	KO Name	KO Description
gene 0016	K01 953	*asnB*	asparagine synthase (glutamine-hydrolysing) [EC:6.3.5.4]
gene 1327	K01 953	*asnB*	asparagine synthase (glutamine-hydrolysing) [EC:6.3.5.4]
gene 0036	K04 072	*adhE*	acetaldehyde dehydrogenase / alcohol dehydrogenase [EC:1.2.1.10 1.1.1.1]
gene 0084	K00 055	-	aryl-alcohol dehydrogenase [EC:1.1.1.90]
gene 0086	K01 426	*amiE*	amidase [EC:3.5.1.4]
gene 0093	K00 020	*mms B*	3-hydroxyisobutyrate dehydrogenase [EC:1.1.1.31]
gene 0128	K01 939	*purA*	adenylosuccinate synthase [EC:6.3.4.4]
gene 0153	K00 826	*ilvE*	branched-chain amino acid aminotransferase [EC:2.6.1.42]
gene 0162	K00 382	*pdhD*	dihydrolipoamide dehydrogenase [EC:1.8.1.4]
gene 0177	K01 733	*thrC*	threonine synthase [EC:4.2.3.1]
gene 0178	K00 003	*hom*	homoserine dehydrogenase [EC:1.1.1.3]
gene 0179	K00 872	*thrB*	homoserine kinase [EC:2.7.1.39]
gene 0207	K23 304	*cysE*	serine O-acetyltransferase [EC:2.3.1.30]
gene 0208	K01 738	*cysK*	cysteine synthase [EC:2.5.1.47]
gene 0251	K01 652	*ilvB*	acetolactate synthase I/II/III large subunit [EC:2.2.1.6]
gene 0258	K01 752	*sdaA*	L-serine dehydratase [EC:4.3.1.17]
gene 0259	K01 752	*sdaA*	L-serine dehydratase [EC:4.3.1.17]
gene 0330	K00 145	*argC*	N-acetyl-gamma-glutamyl-phosphate reductase [EC:1.2.1.38]
gene 0331	K00 620	*argJ*	glutamate N-acetyltransferase / amino-acid N-acetyltransferase [EC:2.3.1.35 2.3.1.1]
gene	K00	*argB*	acetylglutamate kinase [EC:2.7.2.8]

0332	930		
gene 0333	K00 821	*argD*	acetylornithine/N-succinyldiaminopimelate aminotransferase [EC:2.6.1.11 2.6.1.17]
gene 0334	K00 611	*argF*	ornithine carbamoyltransferase [EC:2.1.3.3]
gene 0353	K01 929	*murF*	UDP-N-acetylmuramoyl-tripeptide--D-alanyl-D-alanine ligase [EC:6.3.2.10]
gene 0362	K00 016	*ldh*	L-lactate dehydrogenase [EC:1.1.1.27]
gene 1774	K00 016	*ldh*	L-lactate dehydrogenase [EC:1.1.1.27]
gene 0388	K00 286	*proC*	pyrroline-5-carboxylate reductase [EC:1.5.1.2]
gene 0428	K00 549	*metE*	5-methyltetrahydropteroyltriglutamate--homocysteine methyltransferase [EC:2.1.1.14]
gene 0429	K00 549	*metE*	5-methyltetrahydropteroyltriglutamate--homocysteine methyltransferase [EC:2.1.1.14]
gene 0430	K00 651	*metA*	homoserine O-succinyltransferase/O-acetyltransferase [EC:2.3.1.46 2.3.1.31]
gene 0431	K01 740	*metY*	O-acetylhomoserine (thiol)-lyase [EC:2.5.1.49]
gene 0505	K07 173	*luxS*	S-ribosylhomocysteine lyase [EC:4.4.1.21]
gene 0758	K07 173	*luxS*	S-ribosylhomocysteine lyase [EC:4.4.1.21]
gene 0572	K01 424	*ansA*	L-asparaginase [EC:3.5.1.1]
gene 0630	K05 822	*dapH*	tetrahydrodipicolinate N-acetyltransferase [EC:2.3.1.89]
gene 0631	K05 823	*dapL*	N-acetyldiaminopimelate deacetylase [EC:3.5.1.47]
gene 0688	K00 789	*metK*	S-adenosylmethionine synthetase [EC:2.5.1.6]
gene 0714	K01 756	*purB*	adenylosuccinate lyase [EC:4.3.2.2]
gene 0723	K23 265	*purQ*	phosphoribosylformylglycinamidine synthase subunit PurQ / glutaminase [EC:6.3.5.3 3.5.1.2]
gene 0725	K00 764	*purF*	amidophosphoribosyltransferase [EC:2.4.2.14]
gene 0863	K01 919	*gshA*	glutamate--cysteine ligase [EC:6.3.2.2]
gene 0879	K01 919	*gshA*	glutamate--cysteine ligase [EC:6.3.2.2]

gene 0873	K00 891	*aroK*	shikimate kinase [EC:2.7.1.71]
gene 0882	K08 968	*msrC*	L-methionine (R)-S-oxide reductase [EC:1.8.4.14]
gene 1006	K00 609	*pyrB*	aspartate carbamoyltransferase catalytic subunit [EC:2.1.3.2]
gene 1008	K01 956	*carA*	carbamoyl-phosphate synthase small subunit [EC:6.3.5.5]
gene 1009	K01 955	*carB*	carbamoyl-phosphate synthase large subunit [EC:6.3.5.5]
gene 1041	K00 600	*glyA*	glycine hydroxymethyltransferase [EC:2.1.2.1]
gene 1072	K00 215	*dapB*	4-hydroxy-tetrahydrodipicolinate reductase [EC:1.17.1.8]
gene 1186	K00 841	*patA*	aminotransferase [EC:2.6.1.-]
gene 1188	K00 817	*hisC*	histidinol-phosphate aminotransferase [EC:2.6.1.9]
gene 1189	K01 523	*hisE*	phosphoribosyl-ATP pyrophosphohydrolase [EC:3.6.1.31]
gene 1190	K01 496	*hisI*	phosphoribosyl-AMP cyclohydrolase [EC:3.5.4.19]
gene 1191	K02 500	*hisF*	imidazole glycerol-phosphate synthase subunit HisF [EC:4.3.2.10]
gene 1192	K01 814	*hisA*	phosphoribosylformimino-5-aminoimidazole carboxamide ribotide isomerase [EC:5.3.1.16]
gene 1193	K02 501	*hisH*	imidazole glycerol-phosphate synthase subunit HisH [EC:4.3.2.10]
gene 1194	K01 693	*hisB*	imidazoleglycerol-phosphate dehydratase [EC:4.2.1.19]
gene 1195	K00 013	*hisD*	histidinol dehydrogenase [EC:1.1.1.23]
gene 1196	K00 765	*hisG*	ATP phosphoribosyltransferase [EC:2.4.2.17]
gene 1197	K02 502	*hisZ*	ATP phosphoribosyltransferase regulatory subunit
gene 1198	K04 486	-	histidinol-phosphatase (PHP family) [EC:3.1.3.15]
gene 1307	K01 586	*lysA*	diaminopimelate decarboxylase [EC:4.1.1.20]
gene 1357	K00 133	*asd*	aspartate-semialdehyde dehydrogenase [EC:1.2.1.11]
gene 1362	K01 641	-	hydroxymethylglutaryl-CoA synthase [EC:2.3.3.10]

gene 1373	K01 714	*dapA*	4-hydroxy-tetrahydrodipicolinate synthase [EC:4.3.3.7]
gene 1400	K01 243	*mtnN*	adenosylhomocysteine nucleosidase [EC:3.2.2.9]
gene 1403	K01 778	*dapF*	diaminopimelate epimerase [EC:5.1.1.7]
gene 1415	K01 928	*murE*	UDP-N-acetylmuramoyl-L-alanyl-D-glutamate--2,6-diaminopi melate ligase [EC:6.3.2.13]
gene 1420	K00 928	*lysC*	aspartate kinase [EC:2.7.2.4]
gene 1422	K00 928	*lysC*	aspartate kinase [EC:2.7.2.4]
gene 1600	K01 915	*glnA*	glutamine synthetase [EC:6.3.1.2]
gene 1735	K01 834	*gpm A*	2,3-bisphosphoglycerate-dependent phosphoglycerate mutase [EC:5.4.2.11]
gene 1874	K01 834	*gpm A*	2,3-bisphosphoglycerate-dependent phosphoglycerate mutase [EC:5.4.2.11]
gene 1769	K00 262	*gdhA*	glutamate dehydrogenase (NADP+) [EC:1.4.1.4]
gene 1868	K00 135	*gabD*	succinate-semialdehyde dehydrogenase / glutarate-semialdehyde dehydrogenase [EC:1.2.1.16 1.2.1.79 1.2.1.20]
gene 2005	K00 135	*gabD*	succinate-semialdehyde dehydrogenase / glutarate-semialdehyde dehydrogenase [EC:1.2.1.16 1.2.1.79 1.2.1.20]
gene 1875	K14 155	*patB*	cysteine-S-conjugate beta-lyase [EC:4.4.1.13]
gene 1887	K00 824	*dat*	D-alanine transaminase [EC:2.6.1.21]
gene 1896	K17 363	*urdA*	urocanate reductase [EC:1.3.99.33]
gene 1956	K01 755	*argH*	argininosuccinate lyase [EC:4.3.2.1]
gene 1957	K01 940	*argG*	argininosuccinate synthase [EC:6.3.4.5]
gene 1973	K00 147	*proA*	glutamate-5-semialdehyde dehydrogenase [EC:1.2.1.41]
gene 1974	K00 931	*proB*	glutamate 5-kinase [EC:2.7.2.11]
gene 1978	K01 581	*speC*	ornithine decarboxylase [EC:4.1.1.17]
gene 1998	K00 058	*serA*	D-3-phosphoglycerate dehydrogenase / 2-oxoglutarate reductase [EC:1.1.1.95 1.1.1.399]

gene 1999	K00 831	*serC*	phosphoserine aminotransferase [EC:2.6.1.52]
gene 2008	K00 626	*atoB*	acetyl-CoA C-acetyltransferase [EC:2.3.1.9]
gene 2123	K00 547	*mmu M*	homocysteine S-methyltransferase [EC:2.1.1.10]
gene 2143	K00 820	*glmS*	glutamine---fructose-6-phosphate transaminase (isomerizing) [EC:2.6.1.16]
gene 2145	K00 865	*glxK*	glycerate 2-kinase [EC:2.7.1.165]

Table S4. The genes involved in propionate metabolism of *Ligilactobacillus agilis* W70

Gene ID	KO ID	KO Name	KO Description
gene0362	K00016	*ldh*	L-lactate dehydrogenase [EC:1.1.1.27]
gene1774	K00016	*ldh*	L-lactate dehydrogenase [EC:1.1.1.27]
gene0590	K00656	*pflD*	formate C-acetyltransferase [EC:2.3.1.54]
gene0525	K00625	*pta*	phosphate acetyltransferase [EC:2.3.1.8]
gene0708	K00925	*ackA*	acetate kinase [EC:2.7.2.1]
gene1856	K00925	*ackA*	acetate kinase [EC:2.7.2.1]
gene2133	K00925	*ackA*	acetate kinase [EC:2.7.2.1]

Appendix I.

> the partial sequence of the16S rRNA gene of *Ligilactobacillus agilis* W70

CGAACGGGTGAGTAACACGTGGGTAACCTGCCCAAAAGAGGGGGATAACA
CTTGGAAACAGGTGCTAATACCGCATAACCATGATGACCGCATGGTCATTAT
GTAAAAGATGGTTTCGGCTATCACTTTTGGATGGACCCGCGGCGTATTAACT
TGTTGGTGGGGTAACGGCCTACCAAGGTAATGATACGTAGCCGAACTGAGA
GGTTGATCGGCCACATTGGGACTGAGACACGGCCCAAACTCCTACGGGAG
GCAGCAGTAGGGAATCTTCCACAATGGGCGCAAGCCTGATGGAGCAACGC
CGCGTGAGTGAAGAAGGTCTTCGGATCGTAAAACTCTGTTGTTAGAGAAG
AACATGCAGGAGAGTAACTGTTCTTGTATTGACGGTATCTAACCAGAAAGC
CACGGCTAACTACGTGCCAGCAGCCGCGGTAATACGTAGGTGGCAAGCGTT
GTCCGGATTTATTGGGCGTAAAGGGAACGCAGGCGGTCCTTTAAGTCTGAT
GTGAAAGCCTTCGGCTTAACCGAAGAATTGCATTGGAAACTGGAGGACTT
GAGTGCAGAAGAGGAGAGTGGAACTCCATGTGTAGCGGTGAAATGCGTAG
ATATATGGAAGAACACCAGTGGCGAAAGCGGCTCTCTGGTCTGTAACTGAC
GCTGAGGTTCGAAAGTGTGGGTAGCAAACAGGATTAGATACCCTGGTAGTC
CACACCGTAAACGATGAATGCTAAGTGTTGGAGGGTTTCCGCCCTTCAGTG
CTGCAGCTAACGCAATAAGCATTCCGCCTGGGGAGTACGACCGCAAGGTTG
AAACTCAAAGGAATTGACGGGGGCCCGCACAAGCGGTGGAGCATGTGGTT
TAATTCGAAGCAACGCGAAGAACCTTACCAGGTCTTGACATCTTTTGACCA
TCTTAGAGATAAGATTTTCCCTTCGGGGACAAAATGACAGGTGGTGCATGG
CTGTCGTCAGCTCGTGTCGTGAGATGTTGGGTTAAGTCCCGCAACGAGCGC
AACCCTTGTTGTCAGTTGCCAGCATTAAGTTGGGCACTCTGGCGAGACTGC
CGGTGACAAACCGGAGGAAGGTGGGGACGACGTCAAGTCATCATGCCCCT
TATGACCTGGGCTACACACGTGCTACAATGGACGGTACAACGAGTCGCAAA
CTCGCGAGGGCAAGCTAATCTCTTAAAGCCGTTCTCAGTTCGGATTGTAGG
CTGCAACTCGCCTACATGAAGTCGGAATCGCTAGTAATCGCGAATCAGCAT
GTCGCGGTGAATACGTTCCCGGGCCTTGTACACACCGCCCGTCACACCATG
AGAGTTTGTAACACCCAAAGCCGGTGGGGTAACCTTTAGGAGCTAGC

> *gdhA e*ncoded protein sequence of *Ligilactobacillus agilis* W70

MSYVDEVYNRVVAQNPSQPEFHQAVKEVLESLRPVIEADEAKYRKEALLERL
TTPDRQLLFRVSWVDDQGQVQVNNGYRVQFNNAIGPYKGGLRLHPSVYLGII
KFLGFEQVFKNALTSLPIGGAKGGSDFDPKGKSDREIMAFCQSFMTELYKYIG
ADIDVPAGDIGTGGREIGYLFGQYKRLKSTYEGVLTGKGLTFGGSLARTEATG
YGLLYLVDALLKDHGHDLQGKTVTVSGAGNVAIYAIEKAQQLGAKVVTCSDS
TGWVYDPEGVDVALLKEVKEVKRARLTEYAANRPSAVYHEGRGVWTVKTD
VALPCATQNELNLDDAKQLVANGVVAVCEGANMPTTLEATKYLQENKVLFVP
GKASNAGGVAVSALEMSQNSERLAWSFEEVDGKLKDIMVNIYHNIAAAAKK
YGLDGDYVAGANIAGFLKVAEAMEAQGVV

> *glnA e*ncoded protein sequence of *Ligilactobacillus agilis* W70

MAKATYTKDDIRRIAKEEDVKFLRLMFTDLYGTIKNVEVPISQLDKLLDNKL
MFDGSSIDGFVRIEESDMWLYPDLSTWMIFPWGNEHGKVARIICEVYNADRTP
FYGDPRNNLIRILDEMKELGFTDFNIGPEPEFFLFKLDPETGKPTTHLNDHGSY
FDLAPMDLGENCRRDIVLELEKLGFDVEASHHEVAPGQHEVDFKYADALTA
CDNIQTFKLVVKTVARKHGLHATFMPKPLERINGSGMHLNMSLFTKDGNAFF
DENGQEQLSKEAYYFLGGILKHARNFTALTNPTVNSYKRLVPGYEAPVYVAW
SGHNRSPMVRVPVARGASTRLELRSVDPSANPYMAVAAVLAAGLDGLKNKIE

PAQPVDRNIYSMTEDERKAAGIVDLPSTLHNALKAMREDEVIKDALGNHLYS
NFMEGKRLEWDAYRLEVSQWERDQYLEMY
